# Eiger/TNFα-mediated Dilp8 and ROS production coordinate intra-organ growth in *Drosophila*

**DOI:** 10.1371/journal.pgen.1008133

**Published:** 2019-08-19

**Authors:** Juan A. Sanchez, Duarte Mesquita, María C. Ingaramo, Federico Ariel, Marco Milán, Andrés Dekanty

**Affiliations:** 1 Instituto de Agrobiotecnología del Litoral, Consejo Nacional de Investigaciones Científicas y Técnicas (CONICET) Santa Fe, Argentina; 2 Institute for Research in Biomedicine (IRB Barcelona), The Barcelona Institute of Science and Technology, Barcelona, Spain; 3 Facultad de Bioquímica y Ciencias Biológicas, Universidad Nacional del Litoral (UNL), Santa Fe, Argentina; 4 Institucio Catalana de Recerca i Estudis Avançats (ICREA), Barcelona, Spain; Harvard Medical School, Howard Hughes Medical Institute, UNITED STATES

## Abstract

Coordinated intra- and inter-organ growth during animal development is essential to ensure a correctly proportioned individual. The *Drosophila* wing has been a valuable model system to reveal the existence of a stress response mechanism involved in the coordination of growth between adjacent cell populations and to identify a role of the fly orthologue of p53 (Dmp53) in this process. Here we identify the molecular mechanisms used by Dmp53 to regulate growth and proliferation in a non-autonomous manner. First, Dmp53-mediated transcriptional induction of Eiger, the fly orthologue of TNFα ligand, leads to the cell-autonomous activation of JNK. Second, two distinct signaling events downstream of the Eiger/JNK axis are induced in order to independently regulate tissue size and cell number in adjacent cell populations. Whereas expression of the hormone dILP8 acts systemically to reduce growth rates and tissue size of adjacent cell populations, the production of Reactive Oxygen Species—downstream of Eiger/JNK and as a consequence of apoptosis induction—acts in a non-cell-autonomous manner to reduce proliferation rates. Our results unravel how local and systemic signals act concertedly within a tissue to coordinate growth and proliferation, thereby generating well-proportioned organs and functionally integrated adults.

## Author summary

The coordination of growth between the parts of a given developing organ is an absolute requirement for the generation of functionally integrated structures during animal development. Although this question has fascinated biologists for centuries, the molecular mechanisms responsible have remained elusive to date. In this work, we used the developing wing primordium of *Drosophila* to identify the molecular mechanisms and signaling molecules that mediate communication between adjacent cell populations upon a targeted reduction of growth rate. We first present evidence that the activation of Dmp53 in the growth-depleted territory induces the expression of the fly TNF ligand Eiger, which activates the JNK stress signaling pathway in a cell-autonomous manner. While JNK-dependent expression of the systemic hormone dILP8 reduces the growth and final size of adjacent territories, the production of Reactive Oxygen Species downstream of JNK and the apoptotic machinery act locally to regulate the proliferation of adjacent epithelial cells. Our data reveal how different signals, acting both locally and systemically, can regulate tissue growth and cell proliferation in an independent manner to coordinate the tissue size and cell number of different parts of an organ, ultimately giving rise to well-proportioned adult structures.

## Introduction

Coordinated tissue growth is essential for the generation of functionally integrated organs during animal development, as well as for tissue homeostasis during adult life. Although a broad range of genes and pathways regulating growth has been uncovered, the exact mechanisms by which cells within the same tissue maintain tissue homeostasis by responding to stress in a coordinated manner are less understood.

The p53 tumor suppressor regulates the mammalian cell stress response through direct transcriptional activation of specific target genes involved in cell cycle arrest, DNA repair and apoptosis [1]. Recently, several non-cell-autonomous functions of p53 have been reported to be relevant in tissue homeostasis, as well as in tumor suppression [2,3]. In this regard, the activation of p53 in stromal fibroblasts promotes an antitumor microenvironment by impairing the survival and spread of adjacent tumor cells [4–6]. Likewise, reconstituted MCF7 tumors containing p53-deficient fibroblasts develop faster and are more aggressive than their counterparts carrying wild-type fibroblasts [7]. In fact, the modulation of inflammatory cytokine secretion by tumor-mediated activation of p53 has been shown to limit tissue damage [8], inhibit adjacent epithelial cell transformation [4], and promote macrophage-mediated clearance of tumor and apoptotic cells [9,10].

Studies on the role of p53 in the coordination of growth and cell proliferation have benefited from genetically tractable model systems. The single *Drosophila* orthologue of mammalian p53 (Dmp53) has been demonstrated to be essential for tissue and metabolic homeostasis [2]. In addition to conserved functions that promote apoptosis upon stress, Dmp53 participates in non-cell-autonomous responses, regulating apoptosis-induced proliferation [11–13], cell competition [14,15] and adaptive responses to nutrient stress at the organismal level [16]. Previous studies using the *Drosophila* wing as a model system demonstrated that adjacent cell populations within an organ grow in a coordinated manner, buffering local variations in growth rate, thereby maintaining tissue homeostasis and allowing the development of well-proportioned wings [17,18]. Targeted depletion of growth-promoting genes or disruption of the protein biosynthetic machinery in defined regions of the developing wing primordium reduces size of adjacent unperturbed territories [17,19]. Activation of Dmp53 in the slow-growing cell population is required for proper coordination of intra-organ growth, since depletion of Dmp53 uncouples the growth of adjacent cell populations and ultimately gives rise to wings with incorrect proportions [17]. Several molecular mechanisms, involving either a tissue local response [17] or systemic mechanisms coordinating both intra- and inter-organ growth [19–21], have been proposed to underlie Dmp53-dependent growth coordination.

Here we have identified Eiger (Egr), the unique member of TNF superfamily of ligands in *Drosophila* [22,23] as a direct effector of Dmp53 mediating intra-organ growth. We demonstrate that Egr acts through its receptor Grindelwald (Grnd) and Jun N-terminal Kinase (JNK) signaling to reduce the growth and proliferation rates of adjacent cell populations. The inactivation of Egr or JNK signaling in slow-growing cell populations disrupts the coordination of growth among adjacent tissue domains, resulting in unproportioned adult wings. We further demonstrate that the regulation of tissue growth and proliferation rates by Dmp53 can be uncoupled and independently regulated by two distinct mechanisms downstream of Egr-JNK signaling. Whereas dILP8 expression is required to coordinate intra-organ growth and final tissue size, reactive oxygen species (ROS) production downstream of Egr/JNK and as a consequence of apoptosis induction acts in a non-cell-autonomous manner to regulate the proliferation rates of adjacent epithelial cells. Taken together, our results show how local mechanisms along with systemic responses act together to coordinate the growth and proliferation of different parts of an organ.

## Results

### Dmp53 target genes are upregulated upon growth stress

**Fig 1:**
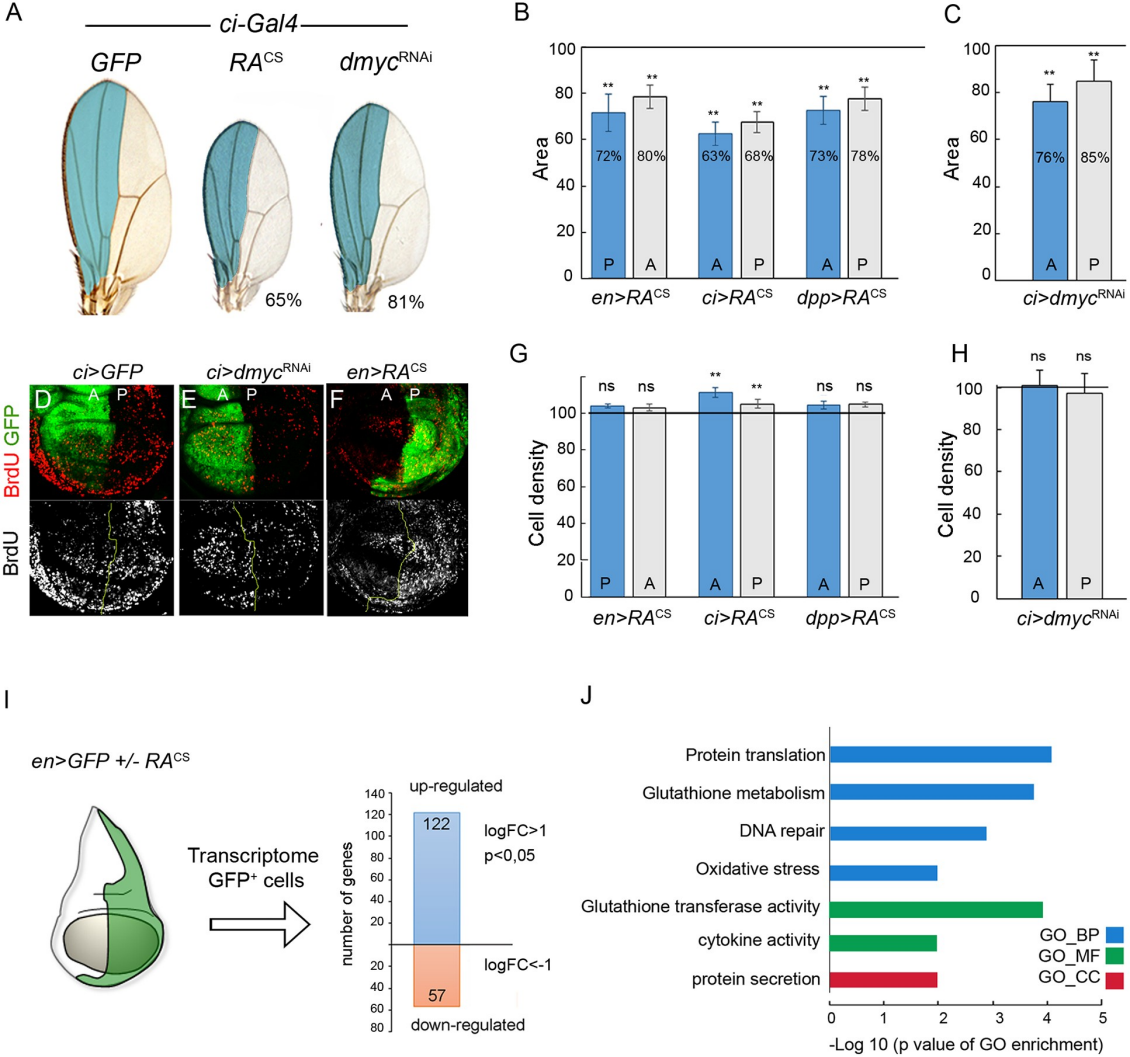
Coordinated intra-organ growth involves the upregulation of Dmp53 target genes. (A) Representative adult wings from individuals expressing GFP, RA^CS^ or *dmyc*^RNAi^ under the control of *ci-Gal4*, which drives expression to the anterior compartment of wing imaginal discs (shown in light blue). Numbers indicate total wing area represented as a percentage of GFP-expressing wings. (B-C, G-H) Histograms plotting normalized area (B-C) or cell density values (G-H) of the anterior (A) and posterior (P) compartments of adult wings from individuals expressing RA^CS^ or *dmyc*^RNAi^ with the indicated Gal4 drivers. Blue bars indicate the transgene-expressing compartment and grey bars indicate the adjacent wild-type compartment. Expression of RA^CS^ or *dmyc*^RNAi^ significantly reduced the size of both transgene-expressing and non-expressing domains. Horizontal line shows the size or cell density values of the normalized control GFP-expressing wings. ** p<0.01. (D-F) BrdU incorporation assay in larval wing discs from individuals expressing GFP, along with the indicated transgenes under the control of *ci-Gal4* or *en-Gal4*. (I) Transcriptome analysis of RNA samples obtained from the P compartment (GFP-positive cells) of wing imaginal discs from individuals expressing GFP or GFP plus RA^CS^ with *en*-*Gal4*. 179 genes were differentially expressed between RA^CS^-expressing and wild-type cells (-1≥fc≥1; p≥0.05). (J) Gene ontology (GO) analysis showed highly enriched GO terms amongst genes upregulated in RA-expressing wing discs. -Log 10 of p-value are shown. GO-BP, biological processes. GO-MF, molecular function. GO-CC, cellular component.

The *Drosophila* wing imaginal disc, a highly proliferative epithelium that grows extensively during larval development, provides an ideal model to study intra-organ growth coordination [24,25]. We made use of the Gal4/UAS system to drive the expression of a cold-sensitive version of type 2 ribosome-inactivating protein Ricin-A (RA^CS^) or an RNAi for the growth-promoting transcription factor dMyc (*dmyc*^RNAi^) to specific regions in the wing primordium. We targeted expression of these transgenes to the anterior (A) and posterior (P) compartments of the developing wing discs, as these compartments are cell populations that do not mix and they are easily identified in the adult wing. This experimental setting also allowed us to characterize the impact of transgene expression on the size of the transgene-expressing compartment, as well as on the size of the adjacent compartment. We used the *ci-Gal4* or *dpp-Gal4* drivers to express these transgenes in the A compartment and the *engrailed-Gal4* (*en-Gal4*) driver for the same purpose in the P compartment. Consistent with previous reports, targeted expression of RA^CS^ or *dmyc*^RNAi^ to the A or P compartments of the wing disc reduced the size of both transgene-expressing and adjacent wild-type territories (Fig 1A–1C; [17]). This non-autonomous decrease in tissue size was also observed when growth was compromised in other regions of the wing by means of a battery of Gal4 drivers, including wing-specific Gal4 drivers [17]. Along with the decrease in tissue size, a non-autonomous reduction in proliferation rates and final cell number was also observed, as determined by measurements of BrdU incorporation (Fig 1D–1F) and cell density in adult wings (Fig 1G and 1H; [17]).

To identify novel molecules and pathways involved in coordinating intra-organ growth, we performed differential gene expression analysis of wing imaginal discs expressing RA^CS^ (*en-Gal4; UAS-RA*^*CS*^, *UAS-GFP*) and control wing discs expressing GFP (*en-Gal4; UAS-GFP*). Transcriptome analysis of RNA samples from P compartment cells (GFP-positive cells) of larvae of the indicated genotypes identified 179 differentially expressed genes (Fig 1I and S1 Table). Quantitative real-time RT-PCR (qRT-PCR) measurements, which showed consistent upregulation of several of these genes in cells expressing either RA^CS^ or *dmyc*^RNAi^, strongly suggested that these two perturbations elicit a common cellular response (S1A Fig). Gene ontology (GO) analysis comprising the 179 identified genes revealed an enrichment in biological processes associated with cellular responses to DNA damage, oxidation-reduction processes, glutathione metabolism, cytokine signaling and extracellular proteins (Fig 1J). In addition, 57 genes previously described as p53 targets [26,27] were specifically upregulated upon RA^CS^ expression (S1C Fig). Together, these results validate previous reports and strongly support the notion that Dmp53 participates in the coordination of tissue growth.

### Eiger/TNFα is required to coordinate intra-organ growth downstream of Dmp53

**Fig 2:**
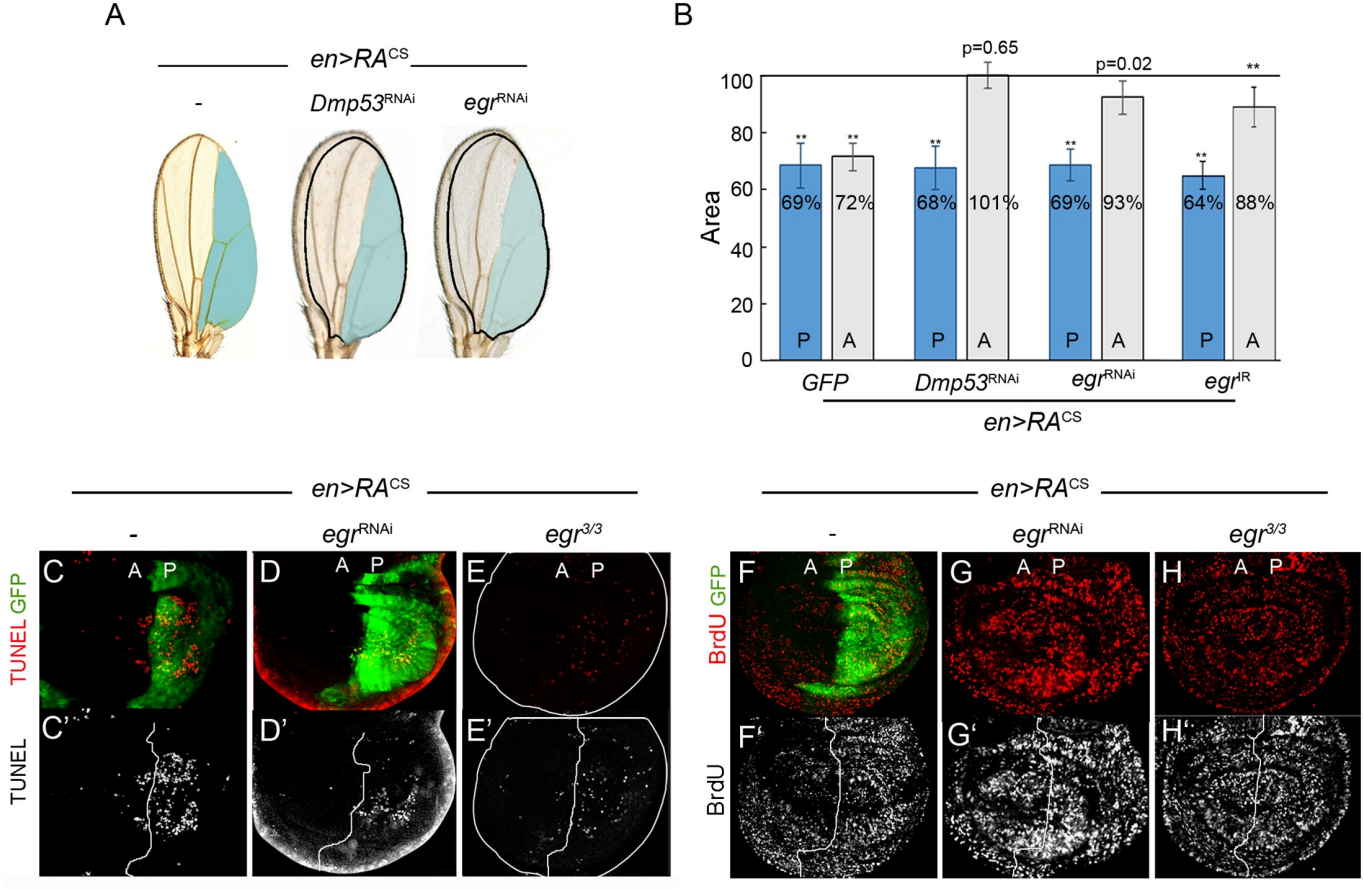
A role of Eiger/TNFα in intra-organ growth. (A) Representative adult wings from individuals expressing the indicated transgenes in posterior compartment (blue) under the control of *en-Gal4*. The expected size of the neighboring domain to give rise to a well-proportioned adult wing is shown. (B) Histogram plotting normalized area of the posterior (P; blue bars) and anterior (A; grey bars) compartments of adult wings from individuals expressing the indicated transgenes with *en-Gal4*. Expression of *Dmp53*^RNAi^, *eiger*^RNAi^ or *eiger*^IR^ reverted the non-autonomous reduction in tissue size caused by RA expression. ** p<0.01. (C-H) Wing discs expressing GFP along with RA^CS^ in the posterior compartment by using *en-Gal4* in different genetic backgrounds (*wild-type* (C,F), co-expressing *eiger*^RNAi^ (D,G) or *eiger*^*3/3*^ (E,H)) and labeled to visualize GFP (green), TUNEL (red in C-E; grey in C’-E’) and BrdU incorporation (red in F-H; grey in F’-H’).

Next, we looked for upregulated secreted signaling molecules in RA^CS^-expressing cells that could mediate tissue non-autonomous responses. GO analysis identified a group of 19 genes coding for extracellular proteins (Fig 1J, S2A Fig and S3 Table). To test whether these molecules and others were actually relevant for intra-organ growth coordination, we performed an *in vivo* loss-of-function screen using *Drosophila* transgenic RNAi lines (S2 Table). Genes encoding for potential secreted factors were specifically silenced in the RA^CS^-expressing domain and screened for their capacity to affect the size of the adjacent compartment in adult wings. Notably, expression of an RNAi form of *eiger (egr)* partially rescued the non-autonomous reduction in tissue size caused by RA^CS^ expression, which resulted in adult wings bearing unproportioned compartments (Fig 2A and 2B). We further confirmed the role of Egr in the non-autonomous regulation of tissue size by using an independent RNAi line (*UAS-egr*^IR^) [22] and *egr* homozygous mutants (Fig 2B and S2B Fig). Of note, the sole expression of *egr*^*RNAi*^ or *Dmp53*^*RNAi*^ had no effect on wing size (S2D Fig).

Egr participates in apoptosis-induced apoptosis, a process by which dying cells within a specific compartment induce apoptosis in the adjacent ones [28]. RA^CS^ expression in *Drosophila* wing discs elicited apoptosis in both transgene-expressing and adjacent wild-type territories (Fig 2C; [17,29]). We then assessed the functional requirement of Egr for apoptosis induction. In this regard, RA^CS^-induced apoptosis was strongly suppressed in both wing disc compartments when Egr was depleted (Fig 2D and 2E). Apoptosis is not required for wing size reduction, but it plays an essential role in the non-autonomous decrease of proliferation rates [17]. We then studied whether Egr is also responsible for reducing proliferation rates upon RA^CS^ expression. Interestingly, *egr*^RNAi^ expression largely rescued the non-autonomous effects of RA on both BrdU incorporation levels and number of mitotic—PH3-positive—cells (Fig 2F and 2G and S2C Fig). Similar results were observed in homozygous *egr*^*3*^ mutant animals (Fig 2H). Together, these observations support a fundamental role of Egr in regulating the size and cell number of adjacent tissue domains.

**Fig 3:**
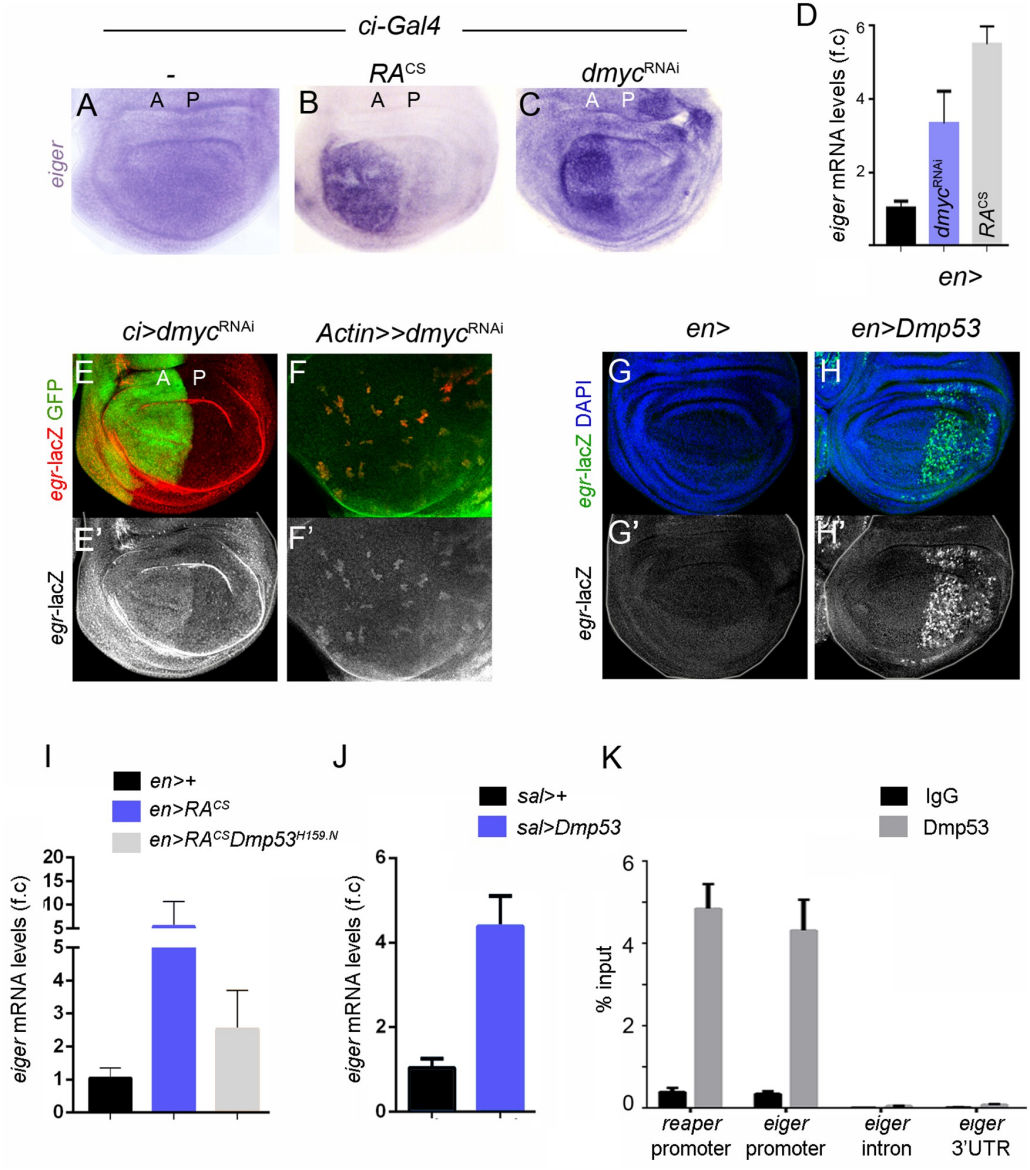
Eiger expression in slow-growing cell populations relies on Dmp53 activity. (A-C) *In situ* hybridization to visualize *eiger* transcript levels in wing discs from individuals expressing GFP (A), RA^CS^ (B) or *dmyc*^RNAi^ (C) under the control of *ci-Gal4*. (D) qRT-PCR showing *eiger* mRNA levels in wing discs expressing RA^CS^ or *dmyc*^*RNAi*^ with *en-Gal4*. Results are expressed as fold induction relative to control wing discs. (E-F) Wing discs carrying *egr*-lacZ transcriptional reporter and stained to visualize GFP (green) and β-gal (red in E-F; grey in E’-F’) protein expression. *dmyc*^RNAi^ is expressed in anterior cells with *ci-Gal4* (E) or in clones using *Actin-Flipout-Gal4* (F). Note increased levels of *egr*-lacZ in dMyc-depleted cells. (G-H) Wing imaginal discs from controls (G) or expressing Dmp53 (H) under the control of *en-Gal4*; *tub*-*Gal80*^*ts*^ and stained to visualize *egr*-lacZ (green or grey) and DAPI (blue). (I-J) qRT-PCR showing *eiger* mRNA levels in wing discs expressing the indicated transgenes under the control of *en-Gal4* (I) or *sal*-*Gal4*;*tub*-*Gal80*^*ts*^ (J). Results are expressed as fold induction with respect to control wing discs. (K) ChIP assays from larvae expressing Dmp53 (*sal-Gal4/+;tub-Gal80*^*ts*^*/UAS*.*Dmp53*) using anti-p53 antibodies or unrelated IgG (control) followed by qPCR for a region overlapping predicted p53-binding elements at *eiger* and *reaper* promoters. *eiger* intronic or 3’UTR regions were used as negative controls. Data (qPCR) are mean ± s.d.

Supporting transcriptome data, qRT-PCR assays showed increased *egr* transcript levels in RA^CS^- and *dmyc*^RNAi^-expressing cells (Fig 3D and S1A Fig). RNA *in situ* hybridization revealed a strong increase in *egr* transcript levels in the RA^CS^- and *dmyc*^RNAi^-expressing wing disc territory (Fig 3A–3C). These findings were confirmed using *in vivo* reporters of both *egr* transcription (*egr*-lacZ; [30]) and Eiger protein expression and localization (Eiger-GFP; [30,31]). *dmyc*^RNAi^-expression in the A compartment (Fig 3E and 3E’), P compartment (S3B Fig) and in clones of cells (Fig 3F and 3F’) showed strong activation of *egr-*lacZ and increased Eiger-GFP levels, thereby indicating that Eiger is specifically and cell-autonomously induced in wing discs upon RA^CS^ or *dmyc*^RNAi^ expression.

We next addressed whether Dmp53 was responsible for the expression of *egr* in RA^CS^-expressing wing discs. Expression of a dominant negative version of Dmp53 lacking DNA-binding activity (Dmp53^H159N^) partially rescued the increase in *egr* expression levels caused by RA^CS^ as well as the induction of known Dmp53 target genes, such as *rpr*, *corp* and *xrp1* (Fig 3I and S3C Fig). We next tested whether Dmp53 overexpression in wing imaginal discs was sufficient to activate *egr* transcription. To avoid the deleterious effects of chronic expression of Dmp53, we used a temperature-sensitive version of Gal80 (Gal80^ts^), a suppressor of Gal4 activity. Expression of Dmp53 for 12 h caused a 4-fold increase in *egr* mRNA levels (Fig 3J, with the use of a wing specific Gal4 driver, *spalt-PE-Gal4*) and activated the expression of *egr*-lacZ in wing discs cells (Fig 3G–3H’). Consistent with recent reports showing Dmp53 binding to *egr* locus [32], chromatin immunoprecipitation (ChIP) assays revealed that Dmp53 binds to predicted *egr* and *reaper* binding sites with similar efficiency (Fig 3K). All together, these results indicate that Dmp53 induces the expression of *egr* through a conserved p53 binding site in its promoter region. Whether Egr activation is also under posttranscriptional regulation (e.g. TACE-mediated cleavage and release of Egr to the extracellular milieu) in the growth-depleted territory and whether this regulation is mediated by Dmp53 are two relevant questions that remain to be explored. Similarly, the experimental observation that Dmp53^H159N^ expression did not completely reduce RA^CS^-induced *egr* expression points to Dmp53-independent inputs into the regulation of *egr* expression upon growth depletion.

### Eiger-induced JNK signaling is required for non-cell-autonomous reduction of growth and proliferation rates

**Fig 4:**
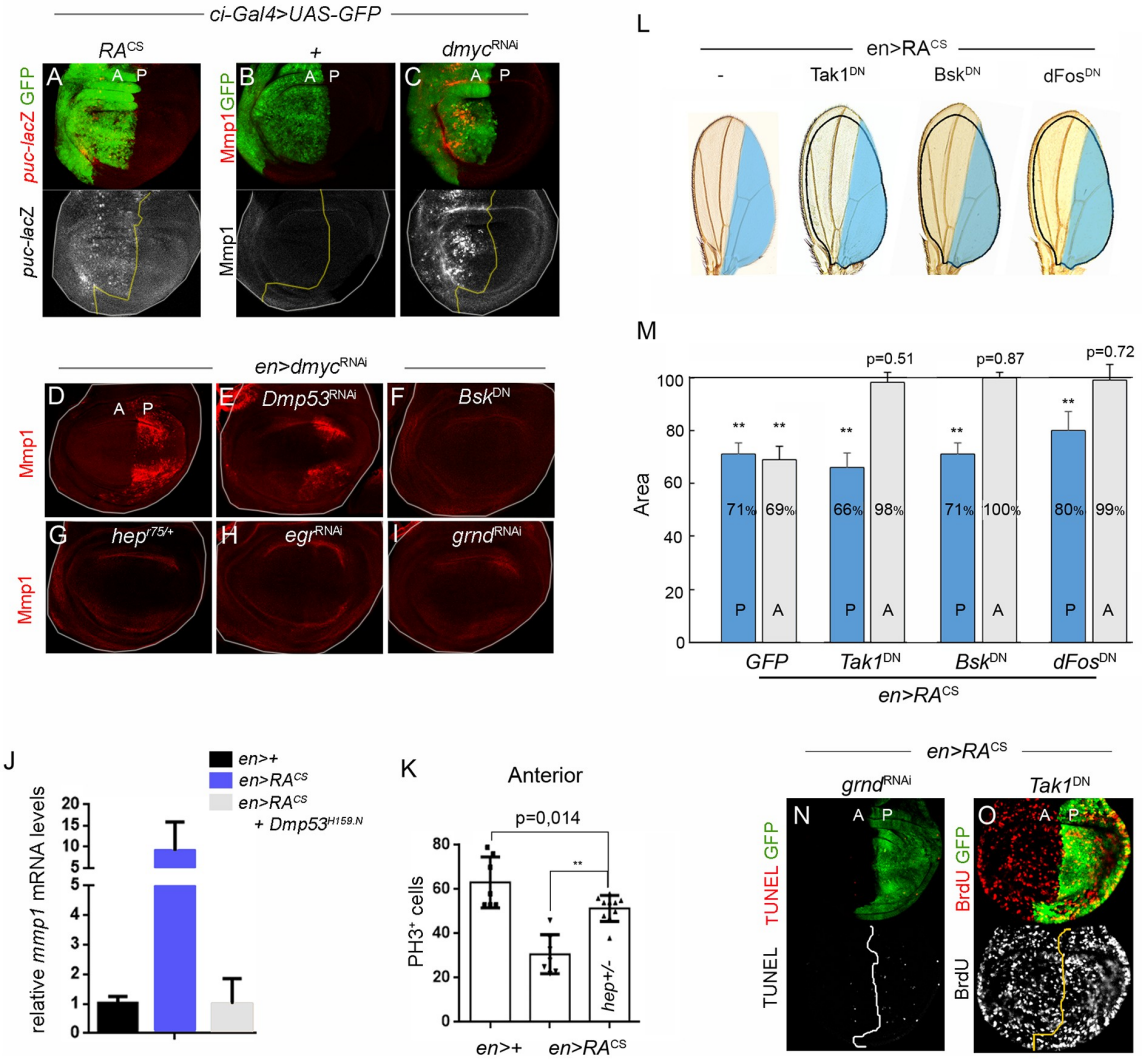
A role of JNK signaling in intra-organ growth. (A-C) Wing imaginal discs from individuals expressing GFP, RA^CS^ or *dmyc*^RNAi^ and stained to visualize GFP (A-C), *puckered*-lacZ (A) and Mmp1 (B-C). (D-I) Wing discs labeled to visualize Mmp1 protein expression from individuals expressing *dmyc*^RNAi^ alone or in combination with *Dmp53*^RNAi^ (E), *Bsk*^DN^ (F), *hep*^*r75/+*^ (G), *egr*^RNAi^ (H) and *grnd*^RNAi^ (I). (J) qRT-PCR showing *mmp1* mRNA levels in wing discs expressing the indicated transgenes under the control of the *en-Gal4* driver. (K) Histogram plotting PH3 positive cells in the anterior (A) compartment of wing imaginal discs from individuals expressing the indicated transgenes under the control of *en-Gal4*. (L) Representative adult wings from individuals expressing the indicated transgenes in posterior compartment (blue) under the control of *en-Gal4*. The expected size of the neighboring domain to give rise to a well-proportioned adult wing is depicted. (M) Histogram plotting normalized area of the posterior (P; blue bars) and anterior (A; grey bars) compartments of adult wings from individuals expressing RA^CS^ along with the indicated transgenes with *en-Gal4*. Blocking JNK pathway by co-expression of Tak^DN^, Bsk^DN^ or Fos^DN^ totally reverted the non-autonomous reduction in tissue size caused by RA expression. ** p<0.01. (N-O) Wing imaginal discs from individuals expressing RA^CS^ along with the indicated transgenes under the control of *en-Gal4* and stained to visualize TUNEL (N) and BrdU incorporation (O).

The JNK cascade is a conserved stress response pathway and an important regulator of tissue growth, proliferation and apoptosis [23,33–38]. Our transcriptome analysis indicated that RA^CS^ expression activates JNK signaling, as we observed the induction of JNK activators such as *egr*, *gadd45* and *traf4*, and JNK target genes such as the JNK-phosphatase *puckered* (*puc*), *matrix metalloproteinase 1* (*mmp1*), *insulin-like-peptide 8* (*dilp8*) and *PDGF- and VEGF-related factor 1* (*Pvf1*) (S1A and S1B and S4A Figs and S3 Table). Consistently, we noted activation of the JNK reporter *puc*-lacZ in wing discs expressing RA^CS^ (Fig 4A) and a significant increase in Mmp1 protein levels in those expressing *dmyc*^RNAi^ (Fig 4B–4D, see also S4B Fig). Mmp1 expression was completely blocked in *hep*^*r75*^ heterozygous animals (Hemipterous; JNK kinase homologue in *Drosophila*) or upon expression of a dominant negative version of Basket (*Bsk*^DN^; JNK homologue in *Drosophila*; Fig 4F and 4G). To test whether the JNK pathway is activated downstream of Egr in slow-growing tissues, we measured Mmp1 protein levels after depletion of Egr or TNFα receptor homologue Grindelwald (Grnd) [39]. Mmp1 ectopic expression caused by *dmyc* knockdown was entirely reverted by expression of either *egr*^RNAi^ or *grnd*^RNAi^ (Fig 4H and 4I). Collectively, these results indicate that Dmp53 activation in growth-depleted territories activates JNK signaling through Egr. Interestingly, expression of the dominant negative version of Dmp53 showed a differential impact on the levels of JNK activity in RA^CS^- and *dmyc*^*RNAi*^-expressing wing discs. While Dmp53 depletion largely reverted RA^CS^-induced expression of Mmp1 (Fig 4J), it caused only a partial reduction of Mmp1 levels in *dmyc*-depleted cells (Fig 4E). These results suggest that JNK is induced in *dmyc*-depleted cells through both Dmp53-dependent and -independent mechanisms.

The observation that Mmp1 and *puckered* expression were restricted to transgene-expressing cell populations suggests that Egr/JNK signaling mediates an autonomous apoptotic response. Indeed, depletion of *grnd* markedly reduced the number of TUNEL-positive cells caused by RA^CS^ expression (Fig 4N). In addition to the expected role of JNK in apoptosis, we questioned whether JNK signaling might contribute to the non-autonomous response of the tissue. To this end, we inhibited JNK signaling specifically in the RA^CS^-expressing compartment and analyzed proliferation rates in adjacent cell populations. Reduced JNK pathway activation, either by using Tak1^DN^ expression or *hep*^*r75*^ mutants, largely rescued the non-autonomous effects of RA^CS^ expression on BrdU incorporation levels and number of mitotic PH3-positive cells (Fig 4K and 4O). We next analyzed the resulting adult wings when activation of the JNK pathway was impaired. Notably, the non-autonomous reduction in tissue size caused by RA expression was completely rescued by the co-expression of Tak1^DN^ or Bsk^DN^ (Fig 4L and 4M). Similar results were obtained using a dominant negative form of dFos, a transcription factor acting downstream of JNK signaling (dFos^DN^, Fig 4L and 4M). The expression of Tak1^DN^, Bsk^DN^ or *grnd*^*RNAi*^ in otherwise *wild-type* wings had no effect on wing size (S4C Fig). Overall, these results indicate that, upon growth depletion, the JNK pathway exerts an essential non-autonomous role in reducing the size of adjacent cell populations.

### Dilp8 is required to coordinate intra-organ growth downstream of Eiger/JNK signaling

**Fig 5:**
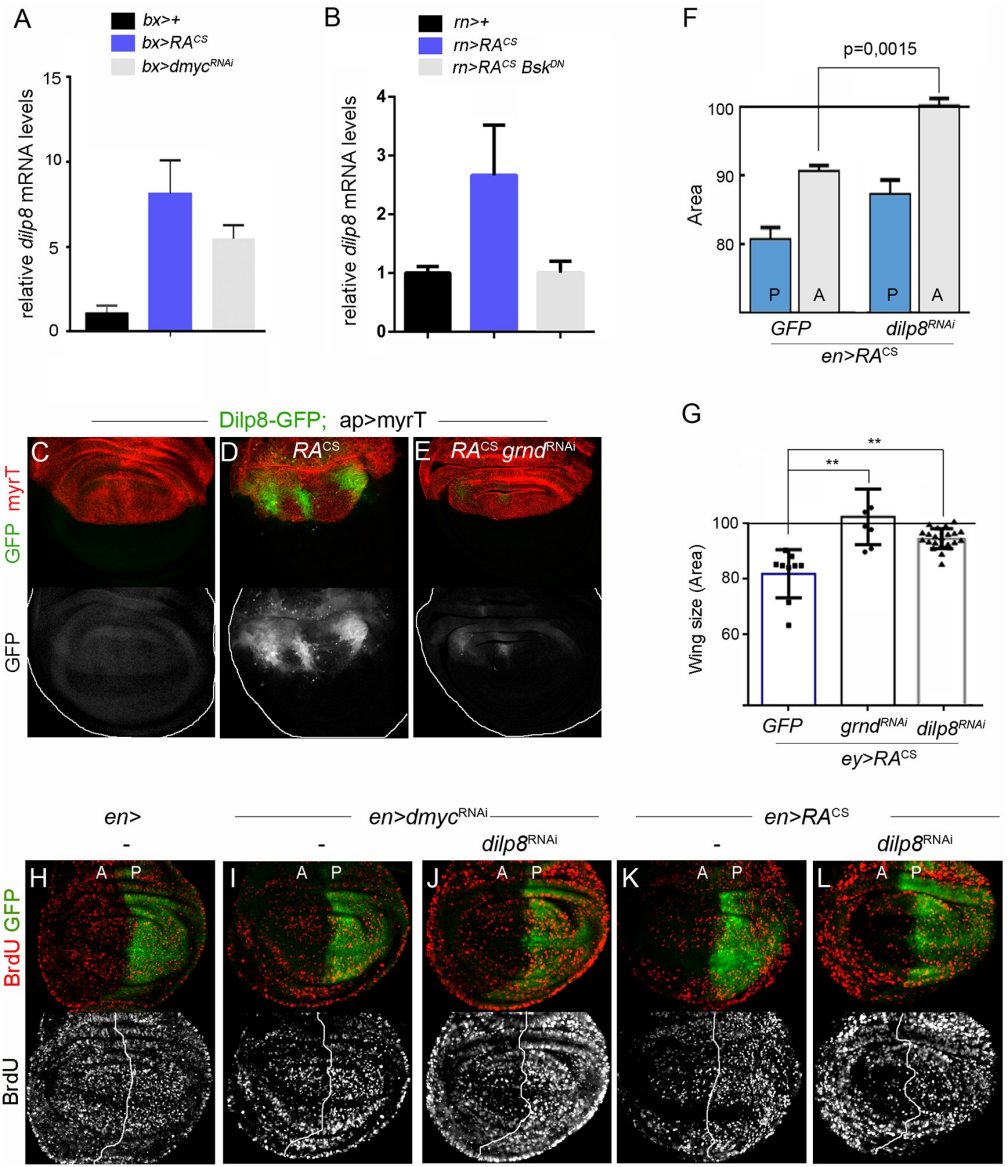
JNK-dependent Dilp8 expression reduces the size of adjacent cell populations. (A-B) qRT-PCR showing *dilp8* transcript levels in wing discs (A) or full larvae (B) expressing the indicated transgenes under the control of *bx-* or *rn*-*Gal4*. Results are expressed as fold induction respect to control wing discs. (C-E) Wing discs carrying a Dilp8-GFP protein trap and expressing the indicated transgenes with *ap-Gal4* (expressed in dorsal compartment) were labeled to visualize GFP (green or grey) and myrTomato (red). (F) Histogram plotting normalized area of the P (blue bars) and A (grey bars) compartments of adult wings from individuals expressing RA^CS^ alone or in combination with *dilp8*^RNAi^ using *en-Gal4*. (G) Histogram plotting normalized wing size from individuals expressing RA^CS^ along with the indicated transgenes with *ey-Gal4* (drives expression to the eye imaginal disc). Horizontal line shows the size of the normalized control wings (*ey-Gal4/UAS-GFP*). (H-L) BrdU incorporation assay in larval wing discs from individuals expressing GFP along with the indicated transgenes under the control of *en-Gal4*. Note the non-autonomous reduction in BrdU incorporation levels caused by *dmyc*^RNAi^ and RA^CS^ expression in Dilp8 knock-down wing discs.

Recently, *Drosophila* insulin-like peptide 8 (Dilp8) has been identified as a signaling molecule produced by imaginal discs in response to tissue damage [40,41]. Dilp8 production by slow-growing or damaged tissues results in activation of neuronal receptor Lgr3, which in turn downregulates the synthesis of Ecdysone, a steroid hormone secreted by the prothoracic gland that, in its active form, stimulates metamorphosis, regulates molting in insects and promotes systemic growth. Consequently, the induction of Dilp8 production by damaged tissues delays metamorphosis and reduces larval and imaginal discs growth [40–44]. Interestingly, we observed strong upregulation of *dilp8* transcript levels upon RA^CS^ or *dmyc*^RNAi^ expression under the control of two different Gal4 drivers expressed in the wing (*bx-Gal4* and *rn-Gal4*; Fig 5A and 5B, see also S1A Fig and S1 Table), and induction of the *dilp8*^MI00727^ protein trap reporter (hereafter Dilp8-GFP, [41]) when the transgene was expressed in the dorsal compartment of the wing with the *ap-Gal4* driver (Fig 5C and 5D).

A number of signaling pathways, including JNK and the bZip DNA binding protein Xrp1, have been shown to regulate Dilp8 expression in response to various types of stress [40,44–46]. The inhibition of JNK, but not Xrp1, caused a significant reduction in the upregulation of *dilp8* mRNA levels caused by RA^CS^ (Fig 5B and S5A Fig). Consistently, Egr/JNK pathway inhibition suppressed RA^CS^-induced Dilp8-GFP expression (Fig 5E and S5D Fig). To examine whether Dilp8 production following RA^CS^ expression is required to reduce the growth of adjacent cell populations, we co-expressed *dilp8*^RNAi^ and RA^CS^ in wing discs using *en-Gal4*, and analyzed adult wing size. Depletion of *dilp8* rescued the non-autonomous reduction in tissue size caused by RA^CS^ expression (Fig 5F). The fact that Dilp8 production affects Ecdysone synthesis point to a systemic, rather than a local, role of this hormone in promoting growth. Indeed, and indicative of systemic mechanisms involved in coordinating inter-organ growth, RA^CS^ expression in eye discs (*ey*>RA^CS^) showed a non-autonomous reduction of adult wing size (Fig 5G) and RNAi-mediated inhibition of Grnd or Dilp8 in slow-growing eye discs fully rescued wing size (Fig 5G). All together, these results indicate that Dilp8 expression downstream of Egr/JNK is required to coordinate organ growth and final tissue size. We next analyzed the contribution of Dilp8 to the non-autonomous reduction of proliferation rates caused by RA^CS^ or *dmyc*^RNAi^. Surprisingly, Dilp8 inhibition did not rescue the non-autonomous reduction in BrdU incorporation levels caused by RA^CS^ or *dmyc*^RNAi^ expression (Fig 5H–5L). However, we noticed that Dilp8 depletion rescued not only the size but also the number of cells in the resulting adult wings (S5C Fig). Taken together, these results point to the existence of signaling molecules regulated by the Dmp53/Egr/JNK axis and acting during larval stages to reduce, in a Dilp8-independent and non-cell autonomous manner, proliferation rates. Our results also suggest the presence of alternative mechanisms, most probably acting in subsequent developmental periods, to adjust final cell number to tissue size.

### Caspase-dependent ROS production downstream of Egr/JNK signaling reduces proliferation rates non-cell-autonomously

**Fig 6:**
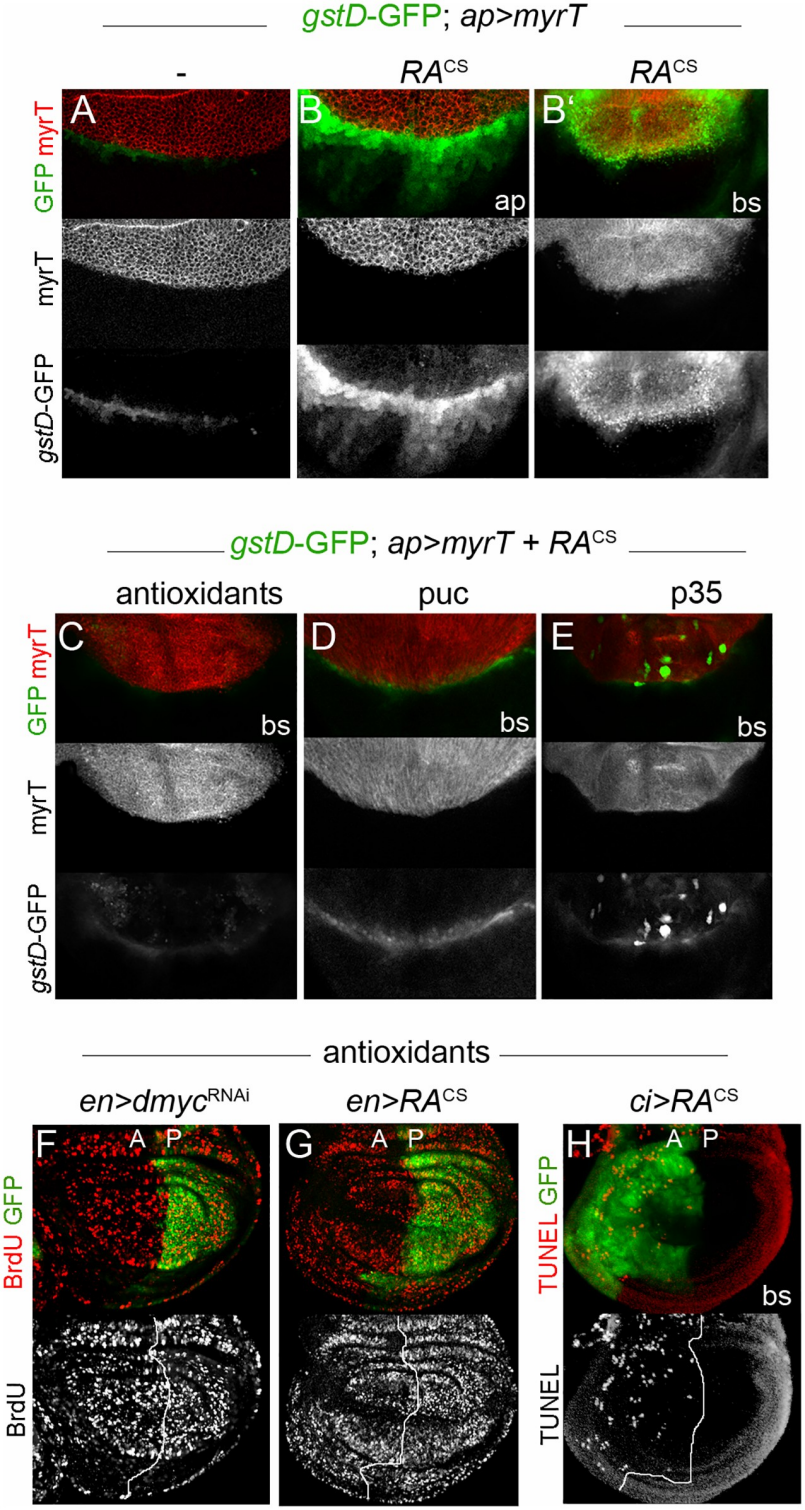
JNK and Caspase-dependent ROS production reduces proliferation rates of adjacent cell populations. (A-E) Wing discs carrying the *gstD1*-GFP reporter and expressing the indicated transgenes with *ap-Gal4* were labeled to visualize GFP (green or grey) and myrTomato (red). RA^CS^ expression showed increased levels of *gstD1*-GFP in both the transgene-expressing and non-expressing domains (B-B’). Antioxidant treatment (C), co-expression of Puckered (puc; D) or p35 (E) fully rescued RA-induced *gstD1*-GFP levels. ap, apical; bs, basal. (F-H) BrdU and TUNEL assays in larval wing discs from individuals expressing GFP along with RA^CS^ or *dmyc*^RNAi^ under the control of *en-* or *ci-Gal4* and cultured in a medium supplemented with antioxidants.

Our GO analysis of RA^CS^-expressing wing discs revealed an enrichment in genes involved in cell redox homeostasis, including the expression of several glutathione S-transferases and other detoxifying genes (Fig 1J and S3 Table), thus suggesting changes in the levels of ROS. To monitor ROS production in developing wing primordia, we used the *gstD1*-GFP reporter, which is activated by ROS in *Drosophila* tissues [47]. *gstD1*-GFP expression was strongly induced not only in the growth-depleted compartment but also in the neighboring compartment following RA^CS^ or *dmyc*^RNAi^ expression (Fig 6A–6B’ and S6A Fig). Whereas *gstD1*-GFP expression was observed mainly at the basal side of the epithelium in the transgene-expressing compartment (Fig 6B’), which is consistent with cellular stress and apoptosis induction, *gstD1*-GFP expression in the neighboring territory was found in intact epithelial cells (Fig 6B). Importantly, RA^CS^-induced *gstD1*-GFP expression was largely rescued by supplementing the medium with antioxidants N-acetylcysteine (NAC), vitamin C and vitamin E (Fig 6C), whereas vehicle treatment had no effect on this parameter (S6A Fig). These observations indicate that ROS produced in the slow-growing cell population spread to the neighboring compartment.

ROS are associated with cellular stress and tissue damage and are important during wound healing and regeneration in many model organisms [48–50]. During *Drosophila* wing regeneration, a burst of ROS generated by dying cells propagates to the nearby surviving tissue, stimulating JNK signaling, which is required for tissue repair [51]. We then addressed whether ROS were produced downstream of the apoptotic pathway in slow-growing compartments. To this end, we inhibited apoptosis in RA^CS^-expressing cells and assessed levels of *gstD1*-GFP expression. Blocking apoptosis by expression of the baculovirus caspase inhibitor p35 largely impaired RA^CS^-induced *gstD1*-GFP expression in both transgene-expressing and adjacent wild-type territories (Fig 6E). Consistent with a role of Egr/JNK signaling upstream of the apoptotic pathway, *gstD1*-GFP was not induced upon expression of the JNK phosphatase Puckered (Fig 6D).

We have previously shown that activation of the apoptotic pathway in slow-growing domains is required to reduce the proliferation rates of adjacent cell populations [17]. Interestingly, the non-autonomous reduction of BrdU incorporation levels caused by RA^CS^ or *dmyc*^RNAi^ expression was largely rescued by supplementing the medium with antioxidants (Fig 6F–6G). Both *gstD1*-GFP expression and proliferation rates were similarly rescued by overexpression of the ROS scavengers’ catalase (Cat) and superoxide dismutase 2 (Sod2) (S6A and S6B Fig). Of note, neither RA^CS^-induced apoptosis (Fig 6H) nor Dilp8-GFP upregulation upon RA^CS^ expression (S5E Fig) were significantly reduced following medium antioxidant supplementation. Collectively, these results indicate that ROS production downstream of Egr/JNK and the apoptotic machinery contributes to the non-autonomous reduction in proliferation rates.

## Discussion

**Fig 7:**
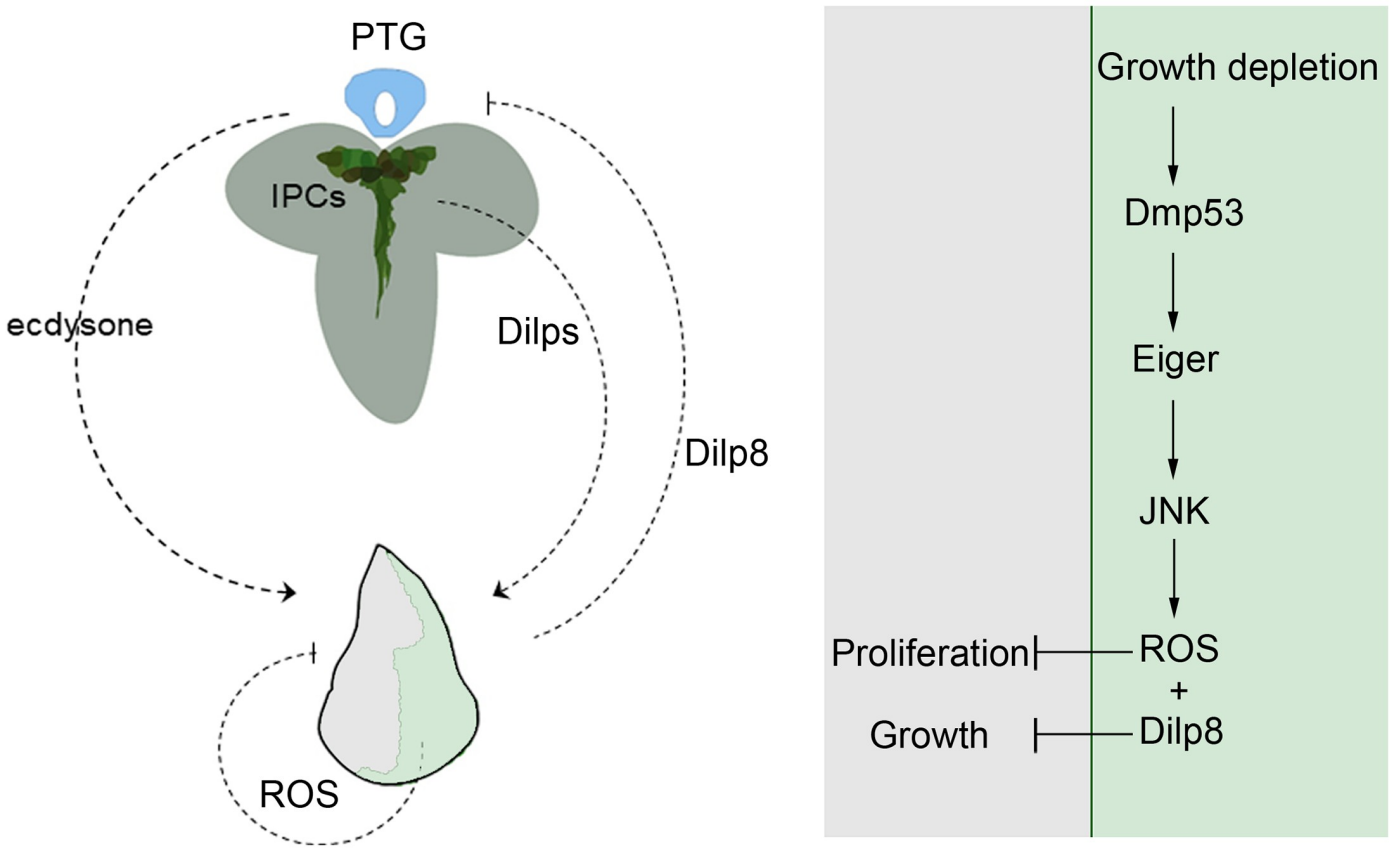
A Dmp53-mediated two-step mechanism regulates growth and proliferation in adjacent populations through the production of systemic and local signals. Targeted expression of RA or depletion of dMyc activates Eiger-JNK signaling downstream of Dmp53. JNK-dependent expression of Dilp8 plays a crucial role in systemically coordinating the size of adjacent cell populations. On the other hand, JNK-dependent ROS production acts locally to regulate proliferation rates in adjacent cell populations.

During animal development, the coordination of intra- and inter-organ growth buffers local growth perturbations and produces individuals with a proper shape and size. Despite the relevance of this process, the molecular mechanisms underlying it are poorly understood. Using the *Drosophila* wing as a model system, we previously demonstrated that impairing growth within defined territories along the wing primordium triggers a Dmp53-dependent reduction of growth and proliferation rates in adjacent non-perturbed cell populations, thus contributing to the generation of smaller but well-proportioned adult wings [17]. Here we provide evidence that Dmp53 regulates intra-organ growth by inducing the expression of the *Drosophila* TNF ligand Eiger [22,23]. We show that Eiger-dependent activation of the JNK pathway in slow-growing compartments is required to reduce the growth and proliferation of adjacent cell populations in a non-cell-autonomous manner. Furthermore, our findings imply that the non-autonomous regulation of tissue growth and proliferation rates can be uncoupled and that they are independently regulated by two distinct molecular mechanisms downstream of Eiger-JNK signaling (Fig 7). On the one hand, Eiger/JNK induces the expression of the relaxin-like Dilp8 protein, which acts systemically to reduce growth in adjacent cell populations without affecting proliferation rates. On the other hand, ROS production downstream of Eiger/JNK acts locally to regulate the proliferation rates of adjacent epithelial cells. In this regard, we show how signals from either neighboring cells or produced at a systemic level play a fundamental role in growth coordination among different parts of an organ (Fig 7).

The ligands of the TNF family are well conserved throughout evolution, and they act mainly through JNK signaling to regulate growth, proliferation and apoptosis [52,53]. *Drosophila* type II transmembrane protein Eiger belongs to this family and exerts intrinsic tumor suppressor activity in epithelia by eliminating oncogenic cells through local endocytic JNK activation [54]. Eiger expression in apoptotic cells activates the JNK pathway in neighboring cells, thus propagating apoptotic JNK signaling along the tissue as part of a process called apoptosis-induced apoptosis [28]. Moreover, it has been reported that interactions between tumor cells and the tumor microenvironment mediated by Eiger and its receptor Grnd also drive JNK activation, tumor growth, and invasive behavior [54–56]. In this work, we showed that Eiger expression in growth-deficient cell populations is required for both apoptotic and proliferative responses in unperturbed territories. Eiger-dependent JNK activation was restricted to slow-growing domains, and inhibition of Grnd-JNK signaling in the growth-depleted territory was sufficient to restore normal size in adjacent domains. Therefore, although Eiger can act as a soluble ligand and activate JNK signaling in distant cells under certain circumstances, the role of this protein in coordinating intra-organ growth seems to be restricted to the growth-depleted tissue and involves local JNK activation.

As mentioned before, the JNK pathway governs proliferation and apoptosis during regeneration and tumorigenesis in *Drosophila* imaginal discs [33–36,38,57]. In addition, JNK signaling regulates organ size during development through a non-canonical, Jun/Fos-independent mechanism [37]. Here we provide evidence that JNK coordinates intra-organ growth in a Fos-dependent manner through a mechanism involving the expression of Dilp8 and generation of ROS, two distinct signaling events that regulate tissue size and cell number. Dilp8, which was recently identified as a signaling molecule produced by damaged imaginal discs, delays metamorphosis and systemically reduces growth [40,41,44]. In developing wing discs, we have shown that Dilp8 expression in slow-growing cell populations contributes to a size reduction of adjacent wild-type territories and thus participates in organ proportion maintenance. The production of Dilp8 by stressed or growth-defective tissues activates Lgr3+ neurons in the central brain to regulate the synthesis of molting hormone ecdysone and/or insulin-like peptides, thereby coordinating growth with developmental timing [43,55,58]. In addition to the role of ecdysone in the regulation of developmental transitions, moderate ecdysone signaling has been shown to promote the growth of imaginal discs both *in vitro* and *in vivo* [59–62]. Indeed, organ growth coordination in response to localized growth defects is mediated, at least in part, by reduced ecdysone levels [19,21]. Remarkably, the coordination of growth between adjacent wing disc compartments can be disrupted by exogenous feeding with 20-hydroxyecdysone (20E) [19]. Therefore, Eiger/JNK-induced Dilp8 expression in the slow-growing compartment most likely regulates growth in a non-autonomous manner via modulation of ecdysone levels.

A number of independent pathways have been shown to activate Dilp8 expression upon several insults, including tissue damage, tissue regeneration, neoplastic growth and impaired ribosomal function [40–42,44–46]. While activation of the JNK and Hippo signaling pathways contributes to the induction of Dilp8 expression in neoplastic tumors, the JAK/STAT pathway drives the expression of this signaling molecule in regenerating tissues. Targeted depletion of ribosomal proteins in developing wing primordia activates Dilp8 expression through the transcription factor Xrp1, and the Xrp1-Dilp8 axis has been shown to have an impact on the growth rates of adjacent cell populations within the wing disc, as well on the growth rates of other primordia [44]. Our data provide evidence of an Xrp1-independent and JNK-dependent mechanism by which Dilp8 expression is activated in growth-depleted territories. These results suggest that distinct signaling pathways, along with a combination of transcription factors, differentially regulate Dilp8 expression in function of the nature of the stress and the cellular context.

In addition to their function in driving apoptosis, ROS also act as signaling molecules, influencing numerous cellular responses, including cell proliferation, differentiation and senescence [63–65]. Although p53 is an important regulator of intracellular ROS levels in vertebrates, downstream mediators of p53 remain to be elucidated [66]. Here we have shown that ROS production downstream of JNK and the apoptotic machinery is required to reduce proliferation rates in *Drosophila* developing epithelial tissues in a non-cell-autonomous manner. In recent reports, ROS were found to be required for apoptosis-induced proliferation and tissue regeneration of *Drosophila* imaginal discs [51,67]. In the first case, extracellular ROS generated by the NADPH oxidase Duox recruit hemocytes to the dying tissue. Hemocytes release Eiger, which promotes JNK activation in epithelial disc cells, thereby driving compensatory proliferation and hyperplastic tissue growth [67]. We found no evidence that hemocytes have a potential analogous role in coordinated intra-organ growth. Our results therefore strongly support the notion that ROS-dependent hemocyte recruitment is context-dependent and that it relies on sustained signals produced by dying cells. Following genetic or physical tissue damage, apoptotic cells generate a burst of ROS that propagates to the nearby surviving cells, thereby stimulating JNK signaling, which is required for cell proliferation and tissue repair [51]. Thus, whereas ROS-stimulated JNK activity promotes cell proliferation in the context of tissue regeneration, JNK-induced ROS reduce proliferation to adjust cell number in a non-cell autonomous manner.

## Materials and methods

### *Drosophila* strains and maintenance

The following *Drosophila* stocks were used: *en-Gal4*, *bx-Gal4*, *rn-Gal4*, *ci-Gal4*, *sal-PE-Gal4* (*sal-Gal4* in the text), *ap-Gal4*, *dpp-Gal4*, *actin-FLPout-Gal4*, *UAS-RA*^*CS*^, *UAS-Bsk*^*DN*^, *UAS-p35*, *UAS-Dmp53*^*H159N*^, *UAS-Dmp53*, *UAS-Tak1*^*K46R*^ (Tak^DN^ in the text), *h*ep^r75^, *UAS-Sod2*, and *UAS-Cat* from the Bloomington Stock Center (USA); and *UAS-eiger*^IR^ [22], *UAS-eiger*^RNAi^, *UAS-grnd*^RNAi^, *UAS-dmyc*^RNAi^, *UAS-Dmp53*^RNAi^, and *Eiger-GFP* (fTRG library) from the Vienna *Drosophila* RNAi Center (VDRC, Austria). The following stocks are described in Flybase: *UAS-eiger*^IR^ [22], *eiger*^*1*^ [22], *eiger*^*3*^ [22], *eiger*-lacZ [30], *UAS-Fos*^*C-Ala*^ (Fos^DN^ in the text [33]). RNAi lines for secreted signaling molecules were obtained from VDRC (Supporting Information S2 Table).

Flies were reared at 25°C on *Drosophila* standard medium (4% glucose, 40 g/L yeast, 1% agar, 25 g/L wheat flour, 25 g/L cornflour, 4 ml/L propionic acid and 1.1 g/L nipagin).

For experiments using the cold-sensitive version of Ricin-A (RA^CS^), embryos containing the Gal4 driver and the *UAS-RA*^*CS*^ transgene were collected over 24 h and maintained at 18°C (restrictive temperature). Early second instar larvae were switched to 29°C (permissive temperature) until late L3 (for wing disc experiments) or until adulthood (for adult wing analysis).

For experiments using the Gal4/Gal80ts system, embryos were collected over 24 h and maintained at 18°C (restrictive temperature). In order to induce transgene expression, 96-h-old larvae were switched to 29°C (permissive temperature) for 12 h before wing disc dissection.

### Immunostaining

Third instar larvae were dissected in cold phosphate-buffered saline (PBS) and fixed in 4% formaldehyde/PBS for 20 min at room temperature. They were then washed and permeabilized in PBT (0.2% Triton X-100 in PBS) for 30 min and blocked in BBT (0.3% BSA, 250 mM NaCl in PBT) for 1 h. Samples were incubated overnight at 4°C with primary antibody diluted in BBT, washed three times (15 min each) in BBT and incubated with secondary antibodies and DAPI (1 μg/ml) for 1.5 hour at room temperature. After three washes with PBT (15 min each), wing discs were placed in mounting medium (80% glycerol/PBS containing 0.05% n-Propyl-Gallate). All steps were performed on a rocking platform at the indicated temperature. The following primary antibodies were used: mouse anti-BrdU (G3G4; Developmental Studies Hybridoma Bank (DSHB)); mouse anti-MMP1 (3A6B4, DSHB); mouse anti-p53 (7A4, DSHB); rabbit anti-p-Histone H3 (sc-8656, Santa Cruz); rabbit anti-β-Gal (A11132, Invitrogen); and sheep anti-Digoxigenin-AP (#11093274910, Roche). The following secondary antibodies were used: anti-mouse IgG-Alexa Fluor 594; anti-mouse IgG-Alexa Fluor 488; anti-rabbit IgG-Alexa Fluor 594; and anti-rabbit IgG-Alexa Fluor 488 (Jackson InmunoResearch).

### TUNEL and BrdU assays

TUNEL was performed as described in [68] using the In Situ Cell Death Detection Kit provided by Roche Diagnostics. BrdU incorporation was performed as described in [68]. Briefly, third-instar larvae were dissected in PBS and incubated with 5-bromo-2’-deoxy-uridine (10 μM, Roche) for 45 min. They were then washed, and fixed in 4% formaldehyde. Following incubation with HCl (2N) for 30 min, samples were neutralized with Borax (100 mM) and immunostained as before.

### Antioxidant treatment

To chemically prevent ROS production, first-instar larvae were transferred to vials containing standard fly food supplemented with the following anti-oxidant concentrations: vitamin E (20 μg/ml); vitamin C (250 μg/ml); and N-acetylcysteine (NAC; 200 μg/ml). Control larvae were transferred in parallel to vials containing standard food supplemented with vehicle. Fresh anti-oxidant stock solutions (10 mg/ml of NAC, diluted in H2O; 2 mg/ml of vitamin E, diluted in absolute ethanol; 25 mg/ml of vitamin E, diluted in H2O) were prepared every week and added to the vials every 48 h.

### Image processing

Images were acquired on a Leica SP8 inverted confocal microscope and analyzed and processed using Fiji [69] and Adobe Photoshop. Wing disc orientation and/or position was adjusted in the field of view for images presented. No relevant information was affected. The original images are available on request.

### RNA in situ hybridization

In situ hybridization was performed as described in [68]. Antisense DIG-labeled *eiger* RNA probe was prepared from XhoI-linearized pBSK-eiger plasmid using T3 RNA polymerase (DIG RNA Labeling Kit, Roche) and detected with the DIG Nucleic Acid Detection Kit (Roche). Wing imaginal discs were mounted in glycerol and imaged with a Nikon E200 bright-field microscope.

### Quantification of adult wing size and cell number

The size of the A and P compartments in adult wings was measured using Fiji. Cell density was measured as the number of hairs (each wing cell differentiates a hair) per defined area, as previously described [17]. At least 10 wings per genotype were scored. Calculated area and cell density values for the different genotypes were normalized to control GFP-expressing wings. Average values and corresponding standard deviation (SD) were calculated, and a two-tailed unpaired Student’s t test was carried out. Calculations and bar graphs were made using the Graph pad Prism 7 software.

### RNA isolation and quantitative RT-PCR

To measure mRNA levels, total RNA was extracted from wing imaginal discs of 30 larvae using TRIZOL RNA Isolation Reagent (Invitrogen). First strand cDNA synthesis was performed using an oligo(dT)18 primer and RevertAid reverse transcriptase (Thermo Scientific) under standard conditions. Quantitative PCR was performed on an aliquot of the cDNA with specific primers (S4 Table) using the StepOnePlus Real-Time PCR System. Expression values were normalized to actin transcript levels. In all cases, three independent samples were collected from each condition and genotype, and duplicate measurements were taken.

### Chromatin immunoprecipitation

ChIP assays were performed following the modEncode protocol [70]. Fifty L3 larvae were dissected for ChIP assays using anti-p53 antibodies (DSHB; 7A4). The primers used to detect immunoprecipitated DNA are listed in S4 Table.

### Microarray

Transcription profiles were obtained at the Functional Genomics Core of IRB Barcelona, as previously described [71]. Three independent RNA samples were prepared from wing imaginal discs of late L3 larvae expressing GFP or GFP plus RA^CS^ under the control of *en-Gal4*. After dissection of wing discs in cold PBS, the posterior GFP-positive compartment was separated under a fluorescence stereoscope and used for RNA extraction with Trizol. Library preparation and amplification were performed using Ovation RNA Amp System V" (Nugen;3100-60). Fragmentation and labelling were performed using Encode Biotin Module (Nugen; 4200–60). The hybridization mixture was prepared following the Affymetrix protocol. Each sample was hybridized to a GeneChip *Drosophila* Genome 2.0 Array (Affymetrix), and then washed and stained in a GeneChip Fluidics Station 450 (Affymetrix). Microarray scanning and CEL fly generation were performed using an Affymetrix GeneChip Scanner GSC3000. To generate log2 expression estimates, overall array intensity was normalized between arrays and the probe intensity of all probes in a probe set was summarized to a single value using gcRMA (gcRMA 2.0.0; Bioconductor package). The transcriptional profile of RA-expressing and non-expressing cells of the posterior compartment was compared, and those genes with fold changes ≥ or ≤ 1 and p value ≤0.05 were considered to be differentially expressed.

Gene ontology (GO) enrichment analysis of those transcripts that were significantly upregulated or downregulated was performed using DAVID v6.7. Microarray datasets have been deposited in the Gene Expression Omnibus (http://www.ncbi.nlm.nih.gov/geo/) under accession number GSE125794.

## Supporting information

S1 Fig (A) qRT-PCR showing transcript levels of a selected group of genes in wing discs expressing RA^CS^ or *myc*^*RNAi*^ with *en-Gal4* or *bx-Gal4*. Results are expressed as fold induction relative to control wing discs. (B) Signaling pathways affected in RA expressing wing discs. (C) Venn diagrams showing overlap between differentially expressed genes in RA-expressing cells and previously identified p53 target genes (Akdemir et al., 2007; van Bergeijk et al., 2012).
(TIF)

S2 Fig (A) List of genes coding for extracellular proteins that were differentially expressed in RA-expressing cells and corresponding fold change. (B) Histogram plotting normalized area and cell density of the anterior (A) compartment of adult wings from individuals expressing RA^CS^ with *hh*-*Gal4* in *eiger*^*3/3*^ mutants. ** p<0.01. (C) Wing imaginal discs from individuals expressing RA^CS^ along with *eiger*^*RNAi*^ under the control of *en-Gal4* and stained to visualize PH3 levels. (D) Histogram plotting normalized area of the anterior (A, grey bars) and posterior (P, blue bars) compartments of adult wings from individuals expressing the indicated transgenes with *en*-*Gal4*.
(TIF)

S3 Fig (A) Expression pattern of *egr*-lacZ [30] and Eiger-GFP [30,31] reporters in the eye and wing imaginal discs of wild-type larvae. (B) Wing discs carrying Eiger-GFP protein trap and stained to visualize GFP (green). *dmyc*^*RNAi*^-expressing cells displayed increased levels of Eiger-GFP. (C) qRT-PCR plotting *rpr*, *corp*, *xrp1* and *damm* mRNA levels in wing discs expressing RA^CS^ or RA^CS^ plus Dmp53^H159N^ with *en-Gal4*. Results are expressed as fold induction respect to control wing discs (en>+).
(TIF)

S4 Fig (A) qRT-PCR showing *mmp1* and *puc* mRNA levels in wing discs expressing the indicated transgenes under the control of *en-Gal4* relative to control wing discs (en>+). (B) Wing discs labeled to visualize Mmp1 protein expression from individuals expressing *dmyc*^RNAi^ under the control of *ap*-*Gal4*. (C-D) Histogram plotting normalized area (C) or density values (D) of the anterior (A, grey bars) and posterior (P, blue bars) compartments of adult wings from individuals expressing the indicated transgenes with *en*-*Gal4*.
(TIF)

S5 Fig (A-B) qRT-PCR showing *dilp8* or *egr* transcript levels in wing discs expressing RA^CS^ or RA^CS^ plus *xrp1*^*RNAi*^ with *bx-Gal4*. Results are expressed as fold induction respect to control wing discs (bx>+). (C) Histogram plotting normalized cell density values of the anterior (A, grey bars) and posterior (P, blue bars) compartments of adult wings from individuals expressing RA^CS^ and *dilp8*^*RNAi*^ with *en*-*Gal4*. (D) Wing discs carrying Dilp8-GFP and expressing the indicated transgenes with *ap-Gal4* were labeled to visualize GFP (green or grey) and myrTomato (red). Expression of Bsk^DN^ largely blocked upregulation of Dilp8-GFP levels observed in dMyc depleted cells. (E) Wing discs carrying Dilp8-GFP and expressing RA^CS^ with *ap-Gal4* were labeled to visualize GFP (green or grey) and myrTomato (red). Upregulation of Dilp8-GFP levels upon RA^CS^ expression was still observed following antioxidant or vehicle treatment.
(TIF)

S6 Fig (A) Wing discs carrying the *gstD1*-GFP reporter and expressing the indicated transgenes with *ap*-*Gal4* were labeled to visualize GFP (green or grey) and myrTomato (red). (B) BrdU incorporation assay in larval wing discs from individuals expressing GFP along with the indicated transgenes under the control of *ci*-*Gal4*. Anti-dMyc staining (green) showed efficiency of gene depletion upon *dmyc*^*RNAi*^ expression.
(TIF)

S1 Table List of genes differentially expressed between RA^CS^-expressing and wild-type cells. (PDF)

S2 Table List of genes encoding for known or predicted secreted molecules and their corresponding transgenic RNAi lines used for the screen. (PDF)

S3 Table Signaling pathways affected in RA^CS^-expressing cells. (PDF)

S4 Table List of primers used in this study. (PDF)

## References

[1] Kruiswijk F, Labuschagne CF, Vousden KH. P53 in Survival, Death and Metabolic Health: a Lifeguard With a Licence To Kill. Nat Rev Mol Cell Biol. Nature Publishing Group; 2015;16: 393–405. 10.1038/nrm4007 26122615

[2] Ingaramo MC, Sánchez JA, Dekanty A. Regulation and function of p53 : a perspective from Drosophila studies. Mech Dev. Elsevier; 2018; 53 10.1016/j.mod.2018.05.007 29800619

[3] Kastenhuber ER, Lowe SW. Putting p53 in Context. Cell. Elsevier Inc.; 2017;170: 1062–1078. 10.1016/j.cell.2017.08.028 28886379 PMC5743327

[4] Lujambio A, Akkari L, Simon J, Grace D, Tschaharganeh DF, Bolden JE, et al Non-cell-autonomous tumor suppression by p53. Cell. Elsevier Inc.; 2013;153: 449–60. 10.1016/j.cell.2013.03.020 23562644 PMC3702034

[5] Hill R, Song Y, Cardiff RD, Van Dyke T. Selective evolution of stromal mesenchyme with p53 loss in response to epithelial tumorigenesis. Cell. 2005;123: 1001–11. 10.1016/j.cell.2005.09.030 16360031

[6] Patocs A, Zhang L, Xu Y, Weber F, Caldes T, Mutter GL, et al Breast-Cancer Stromal Cells with *TP53* Mutations and Nodal Metastases. N Engl J Med. 2007;357: 2543–2551. 10.1056/NEJMoa071825 18094375

[7] Kiaris H, Chatzistamou I, Trimis G, Frangou-Plemmenou M, Pafiti-Kondi A, Kalofoutis A. Evidence for nonautonomous effect of p53 tumor suppressor in carcinogenesis. Cancer Res. 2005;65: 1627–30. 10.1158/0008-5472.CAN-04-3791 15753354

[8] Krizhanovsky V, Yon M, Dickins RA, Hearn S, Simon J, Miething C, et al Senescence of Activated Stellate Cells Limits Liver Fibrosis. Cell. 2008;134: 657–667. 10.1016/j.cell.2008.06.049 18724938 PMC3073300

[9] Xue W, Zender L, Miething C, Dickins RA, Hernando E, Krizhanovsky V, et al Senescence and tumour clearance is triggered by p53 restoration in murine liver carcinomas. Nature. 2007;445: 656–60. 10.1038/nature05529 17251933 PMC4601097

[10] Yoon KW, Byun S, Kwon E, Hwang S-Y, Chu K, Hiraki M, et al Control of signaling-mediated clearance of apoptotic cells by the tumor suppressor p53. Science. 2015;349: 1261669 10.1126/science.1261669 26228159 PMC5215039

[11] Dichtel-Danjoy M-L, Ma D, Dourlen P, Chatelain G, Napoletano F, Robin M, et al Drosophila p53 isoforms differentially regulate apoptosis and apoptosis-induced proliferation. Cell Death Differ. 2013;20: 108–16. 10.1038/cdd.2012.100 22898807 PMC3524635

[12] Wells BS, Yoshida E, Johnston LA. Compensatory proliferation in Drosophila imaginal discs requires Dronc-dependent p53 activity. Curr Biol. 2006;16: 1606–15. 16920621 10.1016/j.cub2006.07.046 PMC1764442

[13] Wells BS, Johnston LA. Maintenance of imaginal disc plasticity and regenerative potential in Drosophila by p53. Dev Biol. Elsevier Inc.; 2012;361: 263–76. 10.1016/j.ydbio.2011.10.012 22036477 PMC3296280

[14] De La Cova C, Senoo-Matsuda N, Ziosi M, Wu DC, Bellosta P, Quinzii CM, et al Supercompetitor status of drosophila Myc cells requires p53 as a Fitness sensor to reprogram metabolism and promote viability. Cell Metab. Elsevier Inc.; 2014;19: 470–483. 10.1016/j.cmet.2014.01.012 24561262 PMC3970267

[15] Kucinski I, Dinan M, Kolahgar G, Piddini E. Chronic activation of JNK JAK/STAT and oxidative stress signalling causes the loser cell status. Nat Commun. Springer US; 2017;8 10.1038/s41467-017-00145-y 28743877 PMC5526992

[16] Barrio L, Dekanty A, Milán M. MicroRNA-Mediated Regulation of Dp53 in the Drosophila Fat Body Contributes to Metabolic Adaptation to Nutrient Deprivation. Cell Rep. 2014;8: 528–541. 10.1016/j.celrep.2014.06.020 25017064

[17] Mesquita D, Dekanty A, Milán M. A dp53-dependent mechanism involved in coordinating tissue growth in Drosophila. PLoS Biol. 2010;8 10.1371/journal.pbio.1000566 21179433 PMC3001892

[18] Garcia-Bellido A, Cortes F, Milan M. Cell interactions in the control of size in Drosophila wings. Proc Natl Acad Sci USA. 1994;91: 10222–10226. 10.1073/pnas.91.21.10222 7937866 PMC44990

[19] Gokhale RH, Hayashi T, Mirque CD, Shingleton AW. Intra-organ growth coordination in Drosophila is mediated by systemic ecdysone signaling. Dev Biol. 2016;418: 135–145. 10.1016/j.ydbio.2016.07.016 27452628

[20] Jaszczak JS, Wolpe JB, Bhandari R, Jaszczak RG, Halme A. Growth Coordination During Drosophila melanogaster Imaginal Disc Regeneration Is Mediated by Signaling Through the Relaxin Receptor Lgr3 in the Prothoracic Gland. Genetics. 2016;204: 703–709. 10.1534/genetics.116.193706 27558136 PMC5068856

[21] Parker NF, Shingleton AW. The coordination of growth among Drosophila organs in response to localized growth-perturbation. Dev Biol. Elsevier B.V.; 2011;357: 318–25. 10.1016/j.ydbio.2011.07.002 21777576

[22] Igaki T, Kanda H, Yamamoto-Goto Y, Kanuka H, Kuranaga E, Aigaki T, et al Eiger, a TNF superfamily ligand that triggers the Drosophila JNK pathway. EMBO J. 2002;21: 3009–18. 10.1093/emboj/cdf306 12065414 PMC126061

[23] Moreno E, Yan M, Basler K. Evolution of TNF signaling mechanisms: JNK-dependent apoptosis triggered by Eiger, the Drosophila homolog of the TNF superfamily. Curr Biol. 2002;12: 1263–8. Available: http://www.ncbi.nlm.nih.gov/pubmed/12176339 12176339 10.1016/s0960-9822(02)00954-5

[24] Juarez-Carreño S, Morante J, Dominguez M. Systemic signalling and local effectors in developmental stability, body symmetry, and size. Cell Stress. 2018;2: 340–361. 10.15698/cst2018.12.167 31225459 PMC6551673

[25] Dekanty A, Milán M. The interplay between morphogens and tissue growth. EMBO Rep. Nature Publishing Group; 2011;12: 1003–10. 10.1038/embor.2011.172 21886183 PMC3185346

[26] Akdemir F, Christich A, Sogame N, Chapo J, Abrams JM. p53 directs focused genomic responses in Drosophila. Oncogene. 2007;26: 5184–93. 10.1038/sj.onc.1210328 17310982

[27] van Bergeijk P, Heimiller J, Uyetake L, Su TT. Genome-wide expression analysis identifies a modulator of ionizing radiation-induced p53-independent apoptosis in Drosophila melanogaster. PLoS One. 2012;7(5):e36539 10.1371/journal.pone.0036539 22666323 PMC3362589

[28] Pérez-Garijo A, Fuchs Y, Steller H. Apoptotic cells can induce non-autonomous apoptosis through the TNF pathway. Elife. 2013;2: e01004 10.7554/eLife.01004 24066226 PMC3779319

[29] Milán M, Campuzano S, García-Bellido A. Developmental parameters of cell death in the wing disc of Drosophila. Proc Natl Acad Sci U S A. 1997;94: 5691–6. 10.1073/pnas.94.11.5691 9159134 PMC20840

[30] Muzzopappa M, Murcia L, Milán M. Feedback amplification loop drives malignant growth in epithelial tissues. Proc Natl Acad Sci. 2017;114: E7291–E7300. 10.1073/pnas.1701791114 28808034 PMC5584413

[31] Sarov M, Barz C, Jambor H, Hein MY, Schmied C, Suchold D, et al A genome-wide resource for the analysis of protein localisation in Drosophila. Elife. 2016;5: e12068 10.7554/eLife.12068 26896675 PMC4805545

[32] Kurtz P, Jones AE, Tiwari B, Link N, Wylie A, Tracy C, et al Drosophila p53 directs non-apoptotic programs in postmitotic tissue. Montell DJ, editor. Mol Biol Cell. 2019; mbc.E18-12-0791. 10.1091/mbc.E18-12-0791 PMC6724604 30892991

[33] Bosch M, Baguñà J, Serras F. Origin and proliferation of blastema cells during regeneration of Drosophila wing imaginal discs. Int J Dev Biol. 2008;52: 1043–50. 10.1387/ijdb.082608mb 18956337

[34] Dekanty A, Barrio L, Muzzopappa M, Auer H, Milán M. Aneuploidy-induced delaminating cells drive tumorigenesis in Drosophila epithelia. Proc Natl Acad Sci U S A. 2012;109: 20549–54. 10.1073/pnas.1206675109 23184991 PMC3528526

[35] Igaki T, Pagliarini RA, Xu T. Loss of cell polarity drives tumor growth and invasion through JNK activation in Drosophila. Curr Biol. 2006;16: 1139–1146. 10.1016/j.cub.2006.04.042 16753569

[36] Uhlirova M, Bohmann D. JNK- and Fos-regulated Mmp1 expression cooperates with Ras to induce invasive tumors in Drosophila. EMBO J. 2006;25: 5294–304. 10.1038/sj.emboj.7601401 17082773 PMC1636619

[37] Willsey HR, Zheng X, Pastor-Pareja J, Willsey AJ, Beachy PA, Xu T. Localized JNK signaling regulates organ size during development. Elife. 2016;5: 1–18. 10.7554/eLife.11491 26974344 PMC4848088

[38] Cosolo A, Jaiswal J, Csordás G, Grass I, Uhlirova M, Classen A-K. JNK-dependent cell cycle stalling in G2 promotes survival and senescence-like phenotypes in tissue stress. Elife. 2019;8: 1–27. 10.7554/elife.41036 30735120 PMC6389326

[39] Andersen DS, Colombani J, Palmerini V, Chakrabandhu K, Boone E, Röthlisberger M, et al The Drosophila TNF receptor Grindelwald couples loss of cell polarity and neoplastic growth. Nature. 2015;522: 482–486. 10.1038/nature14298 25874673

[40] Colombani J, Andersen DS, Léopold P. Secreted peptide Dilp8 coordinates Drosophila tissue growth with developmental timing. Science. 2012;336: 582–5. 10.1126/science.1216689 22556251

[41] Garelli A, Gontijo AM, Miguela V, Caparros E, Dominguez M. Imaginal discs secrete insulin-like peptide 8 to mediate plasticity of growth and maturation. Science. 2012;336: 579–82. 10.1126/science.1216735 22556250

[42] Boone E, Colombani J, Andersen DS, Léopold P. The Hippo signalling pathway coordinates organ growth and limits developmental variability by controlling dilp8 expression. Nat Commun. 2016;7: 13505 10.1038/ncomms13505 27874005 PMC5121414

[43] Vallejo DM, Juarez-Carreño S, Bolivar J, Morante J, Dominguez M. A brain circuit that synchronizes growth and maturation revealed through Dilp8 binding to Lgr3. Science. 2015;350: aac6767. 10.1126/science.aac6767 26429885

[44] Boulan L, Andersen D, Colombani J, Boone E, Léopold P. Inter-Organ Growth Coordination Is Mediated by the Xrp1-Dilp8 Axis in Drosophila. Dev Cell. 2019; 1–8. 10.1016/j.devcel.2019.03.016 31006647

[45] Demay Y, Perochon J, Szuplewski S, Mignotte B, Gaumer S. The PERK pathway independently triggers apoptosis and a Rac1/Slpr/JNK/Dilp8 signaling favoring tissue homeostasis in a chronic ER stress Drosophila model. Cell Death Dis. Nature Publishing Group; 2014;5: e1452–10. 10.1038/cddis.2014.403 25299777 PMC4649510

[46] Katsuyama T, Comoglio F, Seimiya M, Cabuy E, Paro R. During Drosophila disc regeneration, JAK/STAT coordinates cell proliferation with Dilp8-mediated developmental delay. Proc Natl Acad Sci U S A. 2015;112: 2327–36. 10.1073/pnas.1423074112 25902518 PMC4426433

[47] Sykiotis GP, Bohmann D. Keap1/Nrf2 signaling regulates oxidative stress tolerance and lifespan in Drosophila. Dev Cell. 2008;14: 76–85. 10.1016/j.devcel.2007.12.002 18194654 PMC2257869

[48] Schieber M, Chandel NS. ROS Function in Redox Signaling and Oxidative Stress. Curr Biol. 2014;24: R453–R462. 10.1016/j.cub.2014.03.034 24845678 PMC4055301

[49] Niethammer P, Grabher C, Look AT, Mitchison TJ. A tissue-scale gradient of hydrogen peroxide mediates rapid wound detection in zebrafish. Nature. 2009;459: 996–9. 10.1038/nature08119 19494811 PMC2803098

[50] Gauron C, Rampon C, Bouzaffour M, Ipendey E, Teillon J, Volovitch M, et al Sustained production of ROS triggers compensatory proliferation and is required for regeneration to proceed. Sci Rep. 2013;3: 2084 10.1038/srep02084 23803955 PMC3694286

[51] Santabárbara-Ruiz P, López-Santillán M, Martínez-Rodríguez I, Binagui-Casas A, Pérez L, Milán M, et al ROS-Induced JNK and p38 Signaling Is Required for Unpaired Cytokine Activation during Drosophila Regeneration. PLoS Genet. 2015;11: 1–26. 10.1371/journal.pgen.1005595 26496642 PMC4619769

[52] Igaki T, Miura M. The Drosophila TNF ortholog Eiger: emerging physiological roles and evolution of the TNF system. Semin Immunol. Elsevier Ltd; 2014;26: 267–74. 10.1016/j.smim.2014.05.003 24981286

[53] Stronach B. Dissecting JNK signaling, one KKKinase at a time. Dev Dyn. 2005;232: 575–584. 10.1002/dvdy.20283 15704176

[54] Igaki T, Pastor-Pareja JC, Aonuma H, Miura M, Xu T. Intrinsic tumor suppression and epithelial maintenance by endocytic activation of Eiger/TNF signaling in Drosophila. Dev Cell. Elsevier Ltd; 2009;16: 458–65. 10.1016/j.devcel.2009.01.002 19289090 PMC2729686

[55] Colombani J, Andersen DS, Boulan L, Boone E, Romero N, Virolle V, et al Drosophila Lgr3 Couples Organ Growth with Maturation and Ensures Developmental Stability. Curr Biol. 2015;25: 2723–9. 10.1016/j.cub.2015.09.020 26441350

[56] Ohsawa S, Sugimura K, Takino K, Xu T, Miyawaki A, Igaki T. Elimination of Oncogenic Neighbors by JNK-Mediated Engulfment in Drosophila. Dev Cell. 2011;20: 315–328. 10.1016/j.devcel.2011.02.007 21397843

[57] Gerlach SU, Eichenlaub T, Herranz H. Yorkie and JNK Control Tumorigenesis in Drosophila Cells with Cytokinesis Failure. Cell Rep. 2018;23: 1491–1503. 10.1016/j.celrep.2018.04.006 29719260

[58] Garelli A, Heredia F, Casimiro AP, Macedo A, Nunes C, Garcez M, et al Dilp8 requires the neuronal relaxin receptor Lgr3 to couple growth to developmental timing. Nat Commun. 2015;6: 8732 10.1038/ncomms9732 26510564 PMC4640092

[59] Delanoue R, Slaidina M, Léopold P. The steroid hormone ecdysone controls systemic growth by repressing dMyc function in Drosophila fat cells. Dev Cell. 2010;18: 1012–21. 10.1016/j.devcel.2010.05.007 20627082

[60] Jaszczak JS, Wolpe JB, Dao AQ, Halme A. Nitric oxide synthase regulates growth coordination during Drosophila melanogaster imaginal disc regeneration. Genetics. 2015;200: 1219–1228. 10.1534/genetics.115.178053 26081194 PMC4574233

[61] Champlin DT, Truman JW. Ecdysteroid control of cell proliferation during optic lobe neurogenesis in the moth Manduca sexta. Development. 1998;125: 269–77. Available: http://www.ncbi.nlm.nih.gov/pubmed/9486800 9486800 10.1242/dev.125.2.269

[62] Herboso L, Oliveira MM, Talamillo A, Pérez C, González M, Martín D, et al Ecdysone promotes growth of imaginal discs through the regulation of Thor in D. melanogaster. Sci Rep. 2015;5: 12383 10.1038/srep12383 26198204 PMC4510524

[63] Finkel T. Signal transduction by reactive oxygen species. J Cell Biol. 2011;194: 7–15. 10.1083/jcb.201102095 21746850 PMC3135394

[64] Bigarella CL, Liang R, Ghaffari S. Stem cells and the impact of ROS signaling. Development. 2014;141: 4206–4218. 10.1242/dev.107086 25371358 PMC4302918

[65] Owusu-Ansah E, Banerjee U. Reactive oxygen species prime Drosophila haematopoietic progenitors for differentiation. Nature. Nature Publishing Group; 2009;461: 537–41. 10.1038/nature08313 19727075 PMC4380287

[66] Liu D, Xu Y. p53, oxidative stress, and aging. Antioxid Redox Signal. Mary Ann Liebert, Inc.; 2011;15: 1669–78. 10.1089/ars.2010.3644 21050134 PMC3151427

[67] Fogarty CE, Diwanji N, Lindblad JL, Tare M, Amcheslavsky A, Makhijani K, et al Extracellular Reactive Oxygen Species Drive Apoptosis-Induced Proliferation via Drosophila Macrophages. Curr Biol. 2016;26: 575–584. 10.1016/j.cub.2015.12.064 26898463 PMC4765900

[68] Milán M, Campuzano S, García-Bellido A. Cell cycling and patterned cell proliferation in the Drosophila wing during metamorphosis. Proc Natl Acad Sci U S A. 1996;93: 11687–92. 10.1073/pnas.93.21.11687 8876197 PMC38119

[69] Schindelin J, Arganda-Carreras I, Frise E, Kaynig V, Longair M, Pietzsch T, et al Fiji: an open-source platform for biological-image analysis. Nat Methods. Nature Publishing Group; 2012;9: 676–682. 10.1038/nmeth.2019 22743772 PMC3855844

[70] Landt SG, Marinov GK, Kundaje A, Kheradpour P, Pauli F, Batzoglou S, et al ChIP-seq guidelines and practices of the ENCODE and modENCODE consortia. Genome Res. 2012;22: 1813–31. 10.1101/gr.136184.111 22955991 PMC3431496

[71] Clemente-Ruiz M, Murillo-Maldonado JM, Benhra N, Barrio L, Pérez L, Quiroga G, et al Gene Dosage Imbalance Contributes to Chromosomal Instability-Induced Tumorigenesis. Dev Cell. 2016;36: 290–302. 10.1016/j.devcel.2016.01.008 26859353

