# Supplementary figures and images for: Eiger/TNFα-mediated Dilp8 and ROS production coordinate intra-organ growth in Drosophila
Source: PLoS Genet. 2019 Aug 19;15(8):e1008133. doi: 10.1371/journal.pgen.1008133 (PMC6715248; doi:10.1371/journal.pgen.1008133)

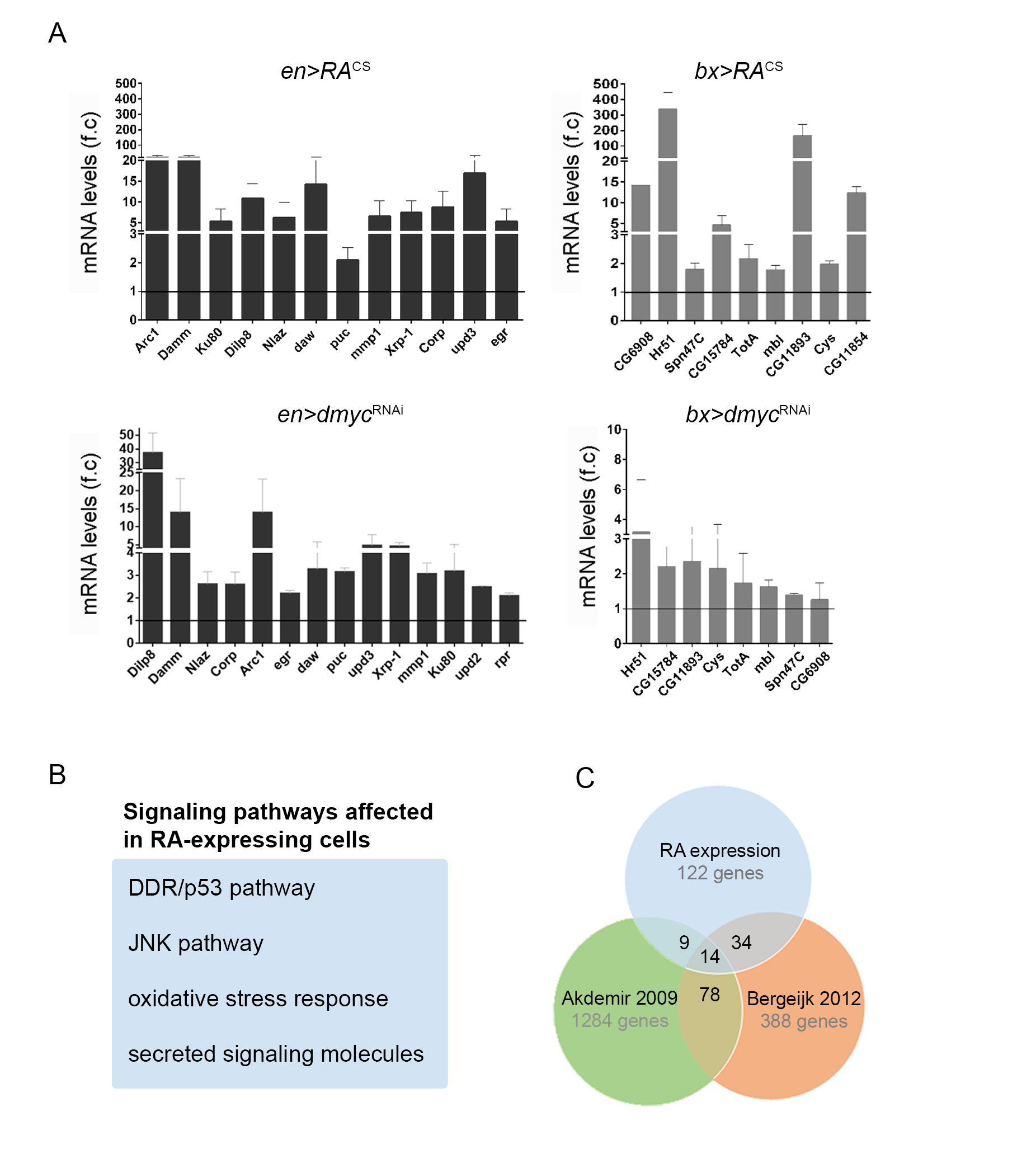

Supplement: S1 Fig — (A) qRT-PCR showing transcript levels of a selected group of genes in wing discs expressing RACS or mycRNAi with en-Gal4 or bx-Gal4. Results are expressed as fold induction relative to control wing discs. (B) Signaling pathways affected in RA expressing wing discs. (C) Venn diagrams showing overlap between differentially expressed genes in RA-expressing cells and previously identified p53 target genes (Akdemir et al., 2007; van Bergeijk et al., 2012). (TIF) [file pgen.1008133.s001.tif]

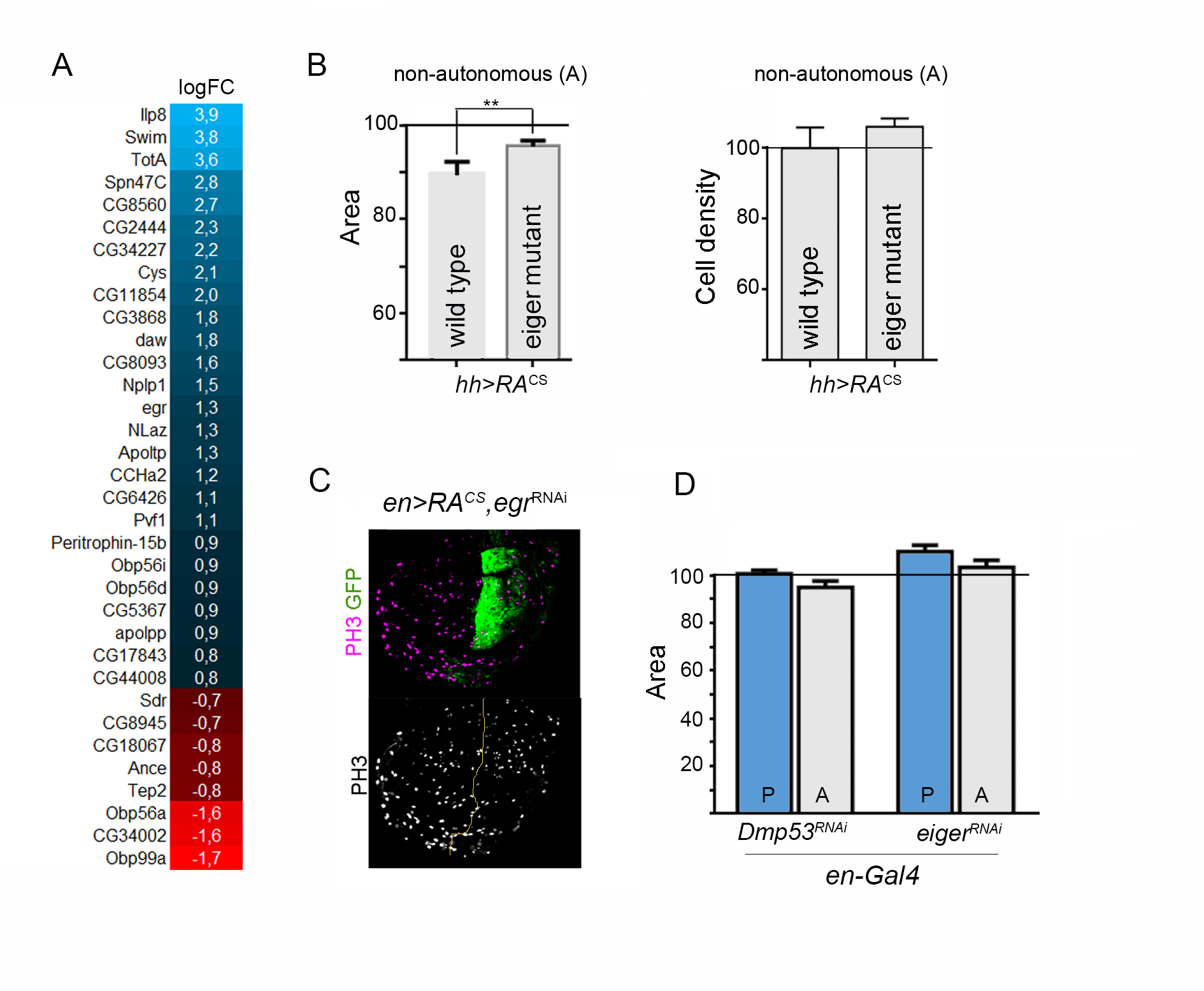

Supplement: S2 Fig — (A) List of genes coding for extracellular proteins that were differentially expressed in RA-expressing cells and corresponding fold change. (B) Histogram plotting normalized area and cell density of the anterior (A) compartment of adult wings from individuals expressing RACS with hh-Gal4 in eiger3/3 mutants. ** p<0.01. (C) Wing imaginal discs from individuals expressing RACS along with eigerRNAi under the control of en-Gal4 and stained to visualize PH3 levels. (D) Histogram plotting normalized area of the anterior (A, grey bars) and posterior (P, blue bars) compartments of adult wings from individuals expressing the indicated transgenes with en-Gal4. (TIF) [file pgen.1008133.s002.tif]

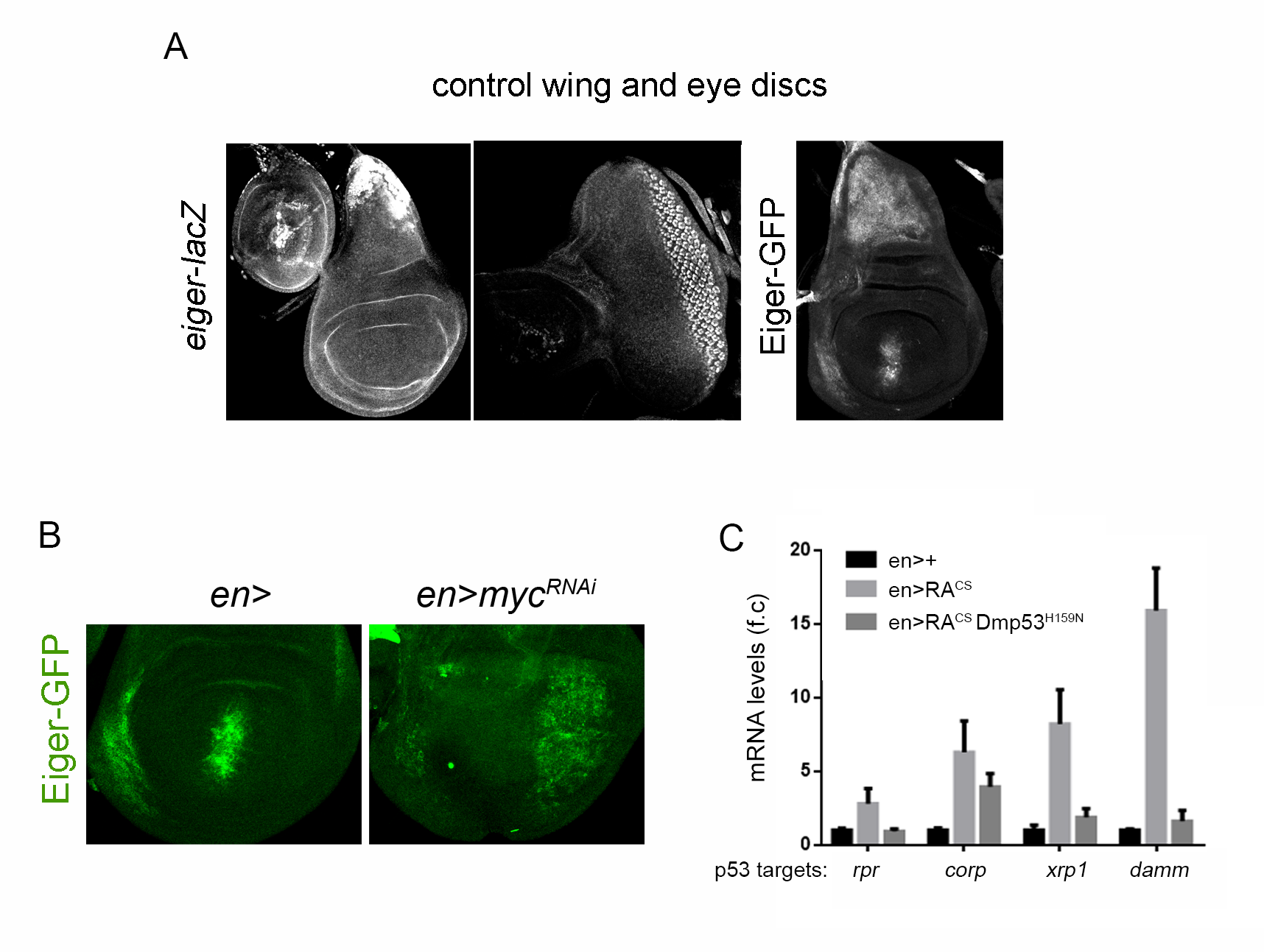

Supplement: S3 Fig — (A) Expression pattern of egr-lacZ [30] and Eiger-GFP [30,31] reporters in the eye and wing imaginal discs of wild-type larvae. (B) Wing discs carrying Eiger-GFP protein trap and stained to visualize GFP (green). dmycRNAi-expressing cells displayed increased levels of Eiger-GFP. (C) qRT-PCR plotting rpr, corp, xrp1 and damm mRNA levels in wing discs expressing RACS or RACS plus Dmp53H159N with en-Gal4. Results are expressed as fold induction respect to control wing discs (en>+). (TIF) [file pgen.1008133.s003.tif]

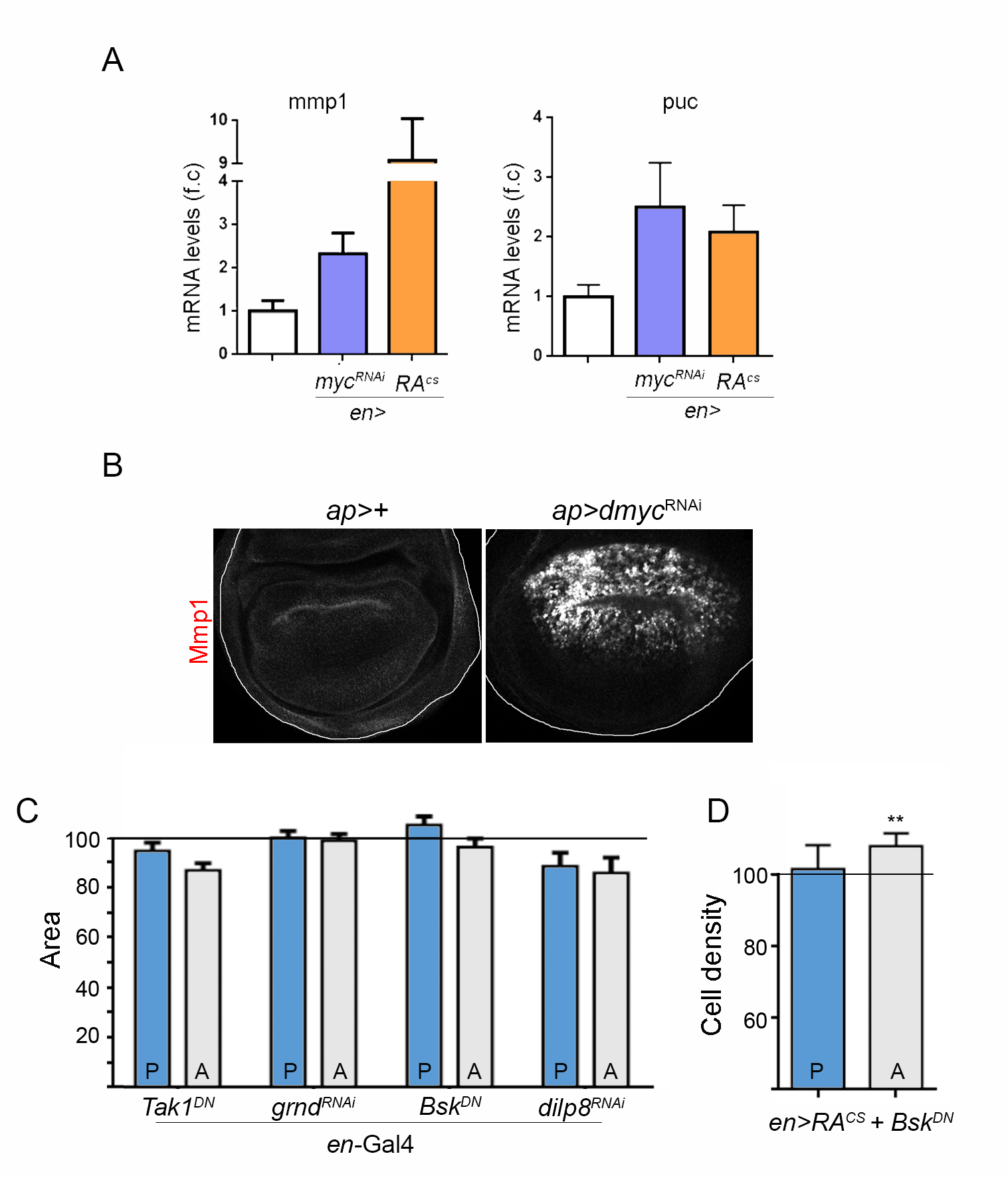

Supplement: S4 Fig — (A) qRT-PCR showing mmp1 and puc mRNA levels in wing discs expressing the indicated transgenes under the control of en-Gal4 relative to control wing discs (en>+). (B) Wing discs labeled to visualize Mmp1 protein expression from individuals expressing dmycRNAi under the control of ap-Gal4. (C-D) Histogram plotting normalized area (C) or density values (D) of the anterior (A, grey bars) and posterior (P, blue bars) compartments of adult wings from individuals expressing the indicated transgenes with en-Gal4. (TIF) [file pgen.1008133.s004.tif]

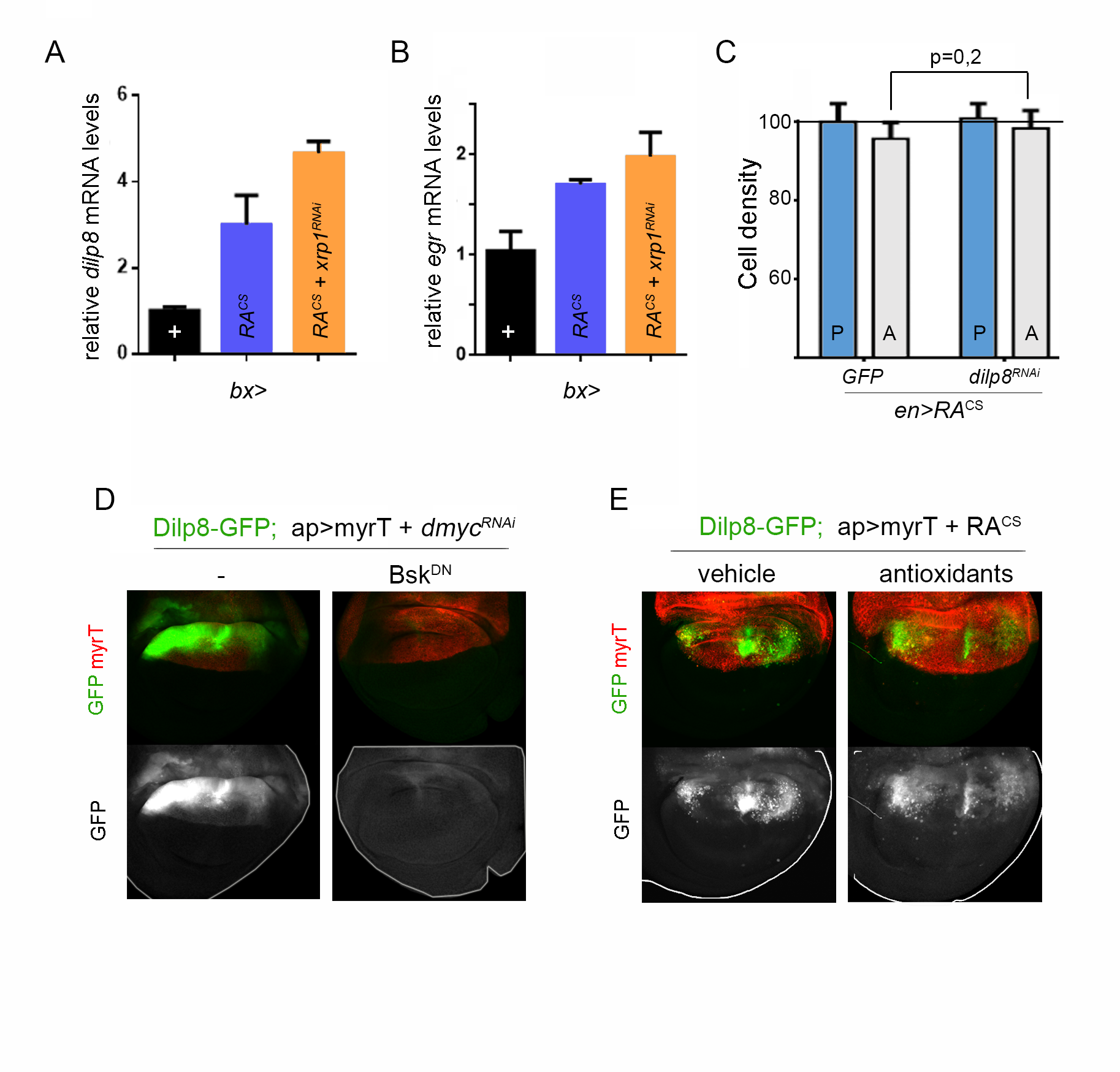

Supplement: S5 Fig — (A-B) qRT-PCR showing dilp8 or egr transcript levels in wing discs expressing RACS or RACS plus xrp1RNAi with bx-Gal4. Results are expressed as fold induction respect to control wing discs (bx>+). (C) Histogram plotting normalized cell density values of the anterior (A, grey bars) and posterior (P, blue bars) compartments of adult wings from individuals expressing RACS and dilp8RNAi with en-Gal4. (D) Wing discs carrying Dilp8-GFP and expressing the indicated transgenes with ap-Gal4 were labeled to visualize GFP (green or grey) and myrTomato (red). Expression of BskDN largely blocked upregulation of Dilp8-GFP levels observed in dMyc depleted cells. (E) Wing discs carrying Dilp8-GFP and expressing RACS with ap-Gal4 were labeled to visualize GFP (green or grey) and myrTomato (red). Upregulation of Dilp8-GFP levels upon RACS expression was still observed following antioxidant or vehicle treatment. (TIF) [file pgen.1008133.s005.tif]

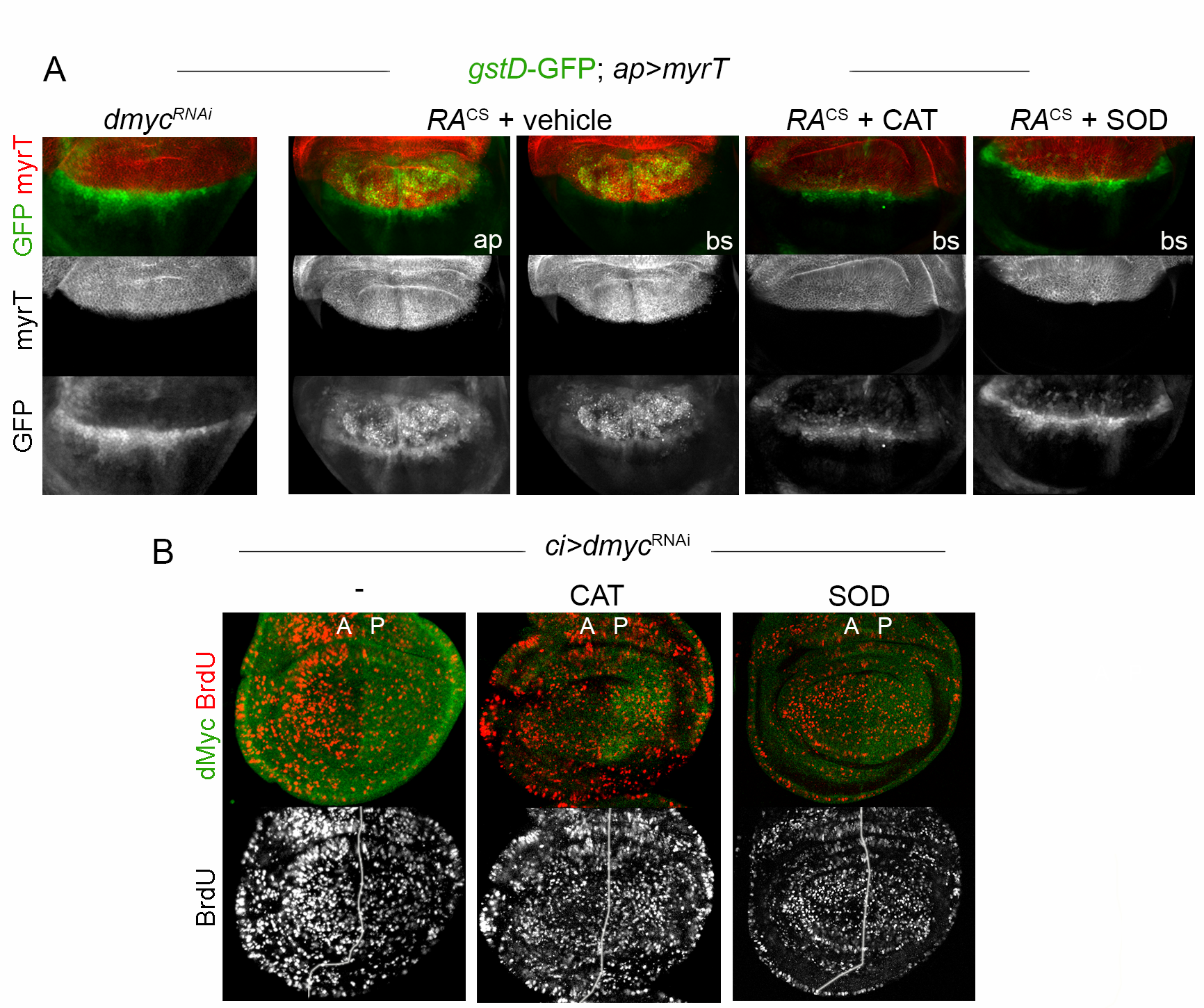

Supplement: S6 Fig — (A) Wing discs carrying the gstD1-GFP reporter and expressing the indicated transgenes with ap-Gal4 were labeled to visualize GFP (green or grey) and myrTomato (red). (B) BrdU incorporation assay in larval wing discs from individuals expressing GFP along with the indicated transgenes under the control of ci-Gal4. Anti-dMyc staining (green) showed efficiency of gene depletion upon dmycRNAi expression. (TIF) [file pgen.1008133.s006.tif]
