# Supplementary material for: Eiger/TNFα-mediated Dilp8 and ROS production coordinate intra-organ growth in Drosophila
Source: PLoS Genet. 2019 Aug 19;15(8):e1008133. doi: 10.1371/journal.pgen.1008133 (PMC6715248; doi:10.1371/journal.pgen.1008133)
Supplement: S1 Table — (PDF) [file pgen.1008133.s007.pdf]

Gene Name	FlyBase ID	Description	logFC	P.Value
CG6908	FBgn0037936	CG6908 gene product from transcript CG6908-RA	9,7	1,4E-06
CG11893	FBgn0039316	CG11893 gene product from transcript CG11893-RA	8,4	5,5E-07
CG14695	FBgn0037850	CG14695 gene product from transcript CG14695-RA	6,7	1,2E-05
CG33468	FBgn0053468	CG33468 gene product from transcript CG33468-RA	6,4	2,6E-05
CG13659	FBgn0039319	CG13659 gene product from transcript CG13659-RA	6,2	3,0E-05
CG31436	FBgn0051436	CG31436 gene product from transcript CG31436-RA	5,7	1,6E-04
Arc1	FBgn0033926	Activity-regulated cytoskeleton associated protein 1	5,4	1,4E-07
Hr51	FBgn0034012	Hormone receptor 51	4,9	4,0E-06
CG42365	FBgn0259711	CG42365 gene product from transcript CG42365-RA	4,3	2,5E-05
CG32368	FBgn0052368	CG32368 gene product from transcript CG32368-RA	4,3	2,5E-04
CG30269	FBgn0050269	CG30269 gene product from transcript CG30269-RB	4,2	1,6E-03
CG12868	FBgn0033945	CG12868 gene product from transcript CG12868-RB	4,1	4,4E-06
Ilp8	FBgn0036690	Insulin-like peptide 8	3,9	6,6E-03
Gp210	FBgn0266580	Gp210 ortholog (H. sapiens)	3,9	1,1E-02
Swim	FBgn0034709	Secreted Wg-interacting molecule	3,8	4,0E-05
CG15784	FBgn0029766	CG15784 gene product from transcript CG15784-RB	3,7	2,7E-05
Sodh-2	FBgn0022359	Sorbitol dehydrogenase-2	3,7	2,0E-03
TotA	FBgn0028396	Turandot A	3,6	1,2E-02
CG33469	FBgn0053469	CG33469 gene product from transcript CG33469-RB	3,6	1,4E-06
CG11897	FBgn0039644	CG11897 gene product from transcript CG11897-RB	3,3	8,8E-04
GstE6	FBgn0063494	Glutathione S transferase E6	3,3	1,4E-05
CG31279	FBgn0051279	CG31279 gene product from transcript CG31279-RD	3,1	1,0E-02
sunn	FBgn0052088	sisters unbound	3,1	1,0E-03
LManII	FBgn0027611	Lysosomal alpha-mannosidase II	2,9	7,0E-03
CG4576	FBgn0038366	CG4576 gene product from transcript CG4576-RA	2,9	7,2E-04
ImpL3	FBgn0001258	Ecdysone-inducible gene L3	2,9	5,6E-04
Ir76a	FBgn0260874	Ionotropic receptor 76a	2,9	5,4E-03
MtnA	FBgn0002868	Metallothionein A	2,9	3,4E-04
Glut1	FBgn0264574	Glucose transporter 1	2,9	1,7E-06
Damm	FBgn0033659	Death associated molecule related to Mch2 caspase	2,8	4,4E-06
Spn47C	FBgn0033574	Serpin 47C	2,8	1,7E-05
CG6678	FBgn0038917	CG6678 gene product from transcript CG6678-RA	2,7	1,7E-02
CG8620	FBgn0040837	CG8620 gene product from transcript CG8620-RA	2,6	3,8E-03
GstD5	FBgn0010041	Glutathione S transferase D5	2,6	4,0E-03
CG12535	FBgn0029657	CG12535 gene product from transcript CG12535-RF	2,5	1,9E-05
CG17018	FBgn0039972	CG17018 gene product from transcript CG17018-RE	2,5	6,3E-03
CG32625	FBgn0052625	CG32625 gene product from transcript CG32625-RA	2,4	5,5E-03
CG32195	FBgn0052195	CG32195 gene product from transcript CG32195-RA	2,4	2,5E-04
CG7768	FBgn0036415	CG7768 gene product from transcript CG7768-RA	2,3	2,8E-04
Gadd45	FBgn0033153	CG11086 gene product from transcript CG11086-RA	2,3	4,0E-04
CG11263	FBgn0036330	CG11263 gene product from transcript CG11263-RA	2,3	2,6E-03
CG34227	FBgn0085256	CG34227 gene product from transcript CG34227-RA	2,2	2,2E-03

CG30424	FBgn0050424	CG30424 gene product from transcript CG30424-RB	2,2	3,6E-03
Rph	FBgn0030230	Rabphilin	2,2	4,7E-02
CG10337	FBgn0032805	CG10337 gene product from transcript CG10337-RA	2,2	4,1E-03
CG31705	FBgn0028490	CG31705 gene product from transcript CG31705-RE	2,1	1,3E-02
Gbeta5	FBgn0030011	CG10763 gene product from transcript CG10763-RA	2,0	4,8E-04
CG31370	FBgn0051370	CG31370 gene product from transcript CG31370-RB	2,0	7,8E-03
Mocs1	FBgn0263241	Molybdenum cofactor synthesis 1 ortholog	2,0	6,0E-04
moody	FBgn0025631	CG4322 gene product from transcript CG4322-RD	2,0	2,5E-02
mbl	FBgn0265487	CG33197 gene product from transcript CG33197-RP	1,9	1,7E-04
CG3568	FBgn0029710	CG3568 gene product from transcript CG3568-RA	1,9	5,1E-03
Tg	FBgn0031975	Transglutaminase	1,9	4,7E-03
CG9171	FBgn0031738	CG9171 gene product from transcript CG9171-RD	1,9	4,1E-04
nAChRalpha4	FBgn0266347	nicotinic Acetylcholine Receptor alpha4	1,8	2,9E-02
unc-13-4A	FBgn0035756	CG32381 gene product from transcript CG32381-RC	1,8	3,4E-02
CG14275	FBgn0032022	CG14275 gene product from transcript CG14275-RA	1,8	1,1E-02
CG3008	FBgn0031643	CG3008 gene product from transcript CG3008-RA	1,8	7,3E-05
CG3448	FBgn0035996	CG3448 gene product from transcript CG3448-RB	1,8	1,5E-03
CG15611	FBgn0034194	CG15611 gene product from transcript CG15611-RA	1,7	7,9E-05
CG31633	FBgn0051633	CG31633 gene product from transcript CG31633-RA	1,7	5,1E-04
CG43117	FBgn0262577	CG43117 gene product from transcript CG43117-RB	1,7	1,3E-03
CG1299	FBgn0035501	CG1299 gene product from transcript CG1299-RA	1,7	3,3E-03
CG2909	FBgn0030189	CG2909 gene product from transcript CG2909-RA	1,6	8,3E-03
CG14879	FBgn0038419	CG14879 gene product from transcript CG14879-RC	1,6	3,6E-02
CG15739	FBgn0030347	CG15739 gene product from transcript CG15739-RA	1,6	4,0E-02
spn-E	FBgn0003483	spindle E	1,6	2,3E-02
Rab9	FBgn0032782	CG9994 gene product from transcript CG9994-RA	1,5	1,5E-03
CG2201	FBgn0032955	CG2201 gene product from transcript CG2201-RF	1,5	3,8E-03
CR42646	FBgn0261429	ncRNA	1,5	2,1E-03
e(y)2b	FBgn0040670	enhancer of yellow 2b	1,5	2,4E-02
her	FBgn0001185	hermaphrodite	1,5	2,5E-02
Cyp6g2	FBgn0033696	CG8859 gene product from transcript CG8859-RA	1,5	3,6E-03
Arc2	FBgn0033928	CG13941 gene product from transcript CG13941-RA	1,4	8,8E-04
CG7924	FBgn0036416	CG7924 gene product from transcript CG7924-RA	1,4	4,4E-02
Ror	FBgn0010407	CG4926 gene product from transcript CG4926-RA	1,4	4,3E-02
CG32196	FBgn0052196	CG32196 gene product from transcript CG32196-RC	1,4	3,1E-03
CHKov2	FBgn0039328	CG10675 gene product from transcript CG10675-RA	1,4	5,8E-04
egr	FBgn0033483	eiger	1,3	1,1E-02
CG7997	FBgn0034117	CG7997 gene product from transcript CG7997-RB	1,3	1,5E-02
Ugt86Di	FBgn0040251	CG6658 gene product from transcript CG6658-RB	1,3	7,3E-04
CG11843	FBgn0039630	CG11843 gene product from transcript CG11843-RB	1,3	1,0E-02
CG13248	FBgn0036984	CG13248 gene product from transcript CG13248-RA	1,3	4,6E-03
FucTC	FBgn0044872	CG40305 gene product from transcript CG40305-RB	1,3	2,9E-02
CG2017	FBgn0037391	CG2017 gene product from transcript CG2017-RD	1,3	4,4E-03

Zmynd10	FBgn0266709	ZMYND10 homolog	1,3	1,3E-02
CG8950	FBgn0034186	CG8950 gene product from transcript CG8950-RB	1,3	1,5E-04
CG7322	FBgn0030968	CG7322 gene product from transcript CG7322-RA	1,3	1,6E-02
lama	FBgn0016031	lamina ancestor	1,3	3,1E-02
CG13255	FBgn0040636	CG13255 gene product from transcript CG13255-RB	1,3	3,3E-03
Ku80	FBgn0041627	CG18801 gene product from transcript CG18801-RA	1,3	7,3E-03
CG17470	FBgn0032869	CG17470 gene product from transcript CG17470-RA	1,2	1,5E-02
Shal	FBgn0005564	Shaker cognate l	1,2	2,3E-02
CG13029	FBgn0036670	CG13029 gene product from transcript CG13029-RC	1,2	2,2E-04
Blos2	FBgn0036118	Biogenesis of lysosome-related organelles complex 1	1,2	7,1E-03
CG6321	FBgn0036117	CG6321 gene product from transcript CG6321-RA	1,2	3,5E-02
plum	FBgn0039431	CG6490 gene product from transcript CG6490-RD	1,2	5,5E-04
GstE7	FBgn0063493	Glutathione S transferase E7	1,2	1,9E-04
CG6272	FBgn0036126	CG6272 gene product from transcript CG6272-RB	1,2	5,4E-04
Gcn5	FBgn0020388	Gcn5 ortholog	1,2	1,1E-02
CG13024	FBgn0036665	CG13024 gene product from transcript CG13024-RC	1,2	1,8E-02
CG32549	FBgn0052549	CG32549 gene product from transcript CG32549-RD	1,2	4,2E-02
CG14545	FBgn0040602	CG14545 gene product from transcript CG14545-RA	1,2	1,0E-02
CG6701	FBgn0033889	CG6701 gene product from transcript CG6701-RD	1,2	2,3E-02
GstE9	FBgn0063491	Glutathione S transferase E9	1,2	3,0E-03
CG13890	FBgn0035169	CG13890 gene product from transcript CG13890-RA	1,1	6,9E-05
CG6426	FBgn0034162	CG6426 gene product from transcript CG6426-RA	1,1	2,7E-02
CG10916	FBgn0034312	CG10916 gene product from transcript CG10916-RB	1,1	4,4E-04
CG3726	FBgn0029824	CG3726 gene product from transcript CG3726-RB	1,1	2,0E-02
CG6762	FBgn0030876	CG6762 gene product from transcript CG6762-RD	1,1	4,9E-02
CG31694	FBgn0051694	CG31694 gene product from transcript CG31694-RA	1,1	1,2E-02
Pvf1	FBgn0030964	PDGF- and VEGF-related factor 1	1,1	4,6E-02
fan	FBgn0028379	farinelli	1,1	4,7E-02
CG42299	FBgn0259195	CG42299 gene product from transcript CG42299-RA	1,1	2,4E-02
Alg-2	FBgn0086378	Apoptosis-linked gene-2	1,1	8,8E-03
CG32645	FBgn0052645	CG32645 gene product from transcript CG32645-RB	1,1	1,1E-02
Ttc19	FBgn0032744	Tetratricopeptide repeat domain 19	1,0	5,8E-03
CG7367	FBgn0031976	CG7367 gene product from transcript CG7367-RD	1,0	1,1E-02
LamC	FBgn0010397	Lamin C	1,0	4,1E-02
CG18094	FBgn0032791	CG18094 gene product from transcript CG18094-RB	1,0	2,3E-02
Gclc	FBgn0040319	Glutamate-cysteine ligase catalytic subunit	1,0	8,5E-04
Xrp1	FBgn0261113	CG17836 gene product from transcript CG17836-RB	1,0	9,4E-04
CG3009	FBgn0029720	CG3009 gene product from transcript CG3009-RD	-1,0	4,6E-02
hd	FBgn0086695	humpty dumpty	-1,0	1,4E-02
CG30099	FBgn0050099	CG30099 gene product from transcript CG30099-RA	-1,0	1,0E-03
CG9850	FBgn0034903	CG9850 gene product from transcript CG9850-RC	-1,0	4,2E-02
Gbs-76A	FBgn0036862	Glycogen binding subunit 76A	-1,0	7,5E-03
CG14598	FBgn0037503	CG14598 gene product from transcript CG14598-RA	-1,1	3,4E-02

kar	FBgn0001296	karmoisin	-1,1	2,3E-02
CG13044	FBgn0036599	CG13044 gene product from transcript CG13044-RA	-1,1	4,7E-03
CG4562	FBgn0038740	CG4562 gene product from transcript CG4562-RC	-1,1	2,5E-02
CG13737	FBgn0036382	CG13737 gene product from transcript CG13737-RA	-1,1	4,6E-02
Sep4	FBgn0259923	Septin 4	-1,1	1,3E-02
mthl4	FBgn0034219	methuselah-like 4	-1,1	2,9E-03
CG10211	FBgn0032685	CG10211 gene product from transcript CG10211-RB	-1,2	4,1E-02
RhoGAP100F	FBgn0039883	Rho GTPase activating protein at 100F	-1,2	2,5E-02
grass	FBgn0039494	Gram-positive Specific Serine protease	-1,2	7,4E-03
CG15356	FBgn0031377	CG15356 gene product from transcript CG15356-RA	-1,2	4,9E-02
CR31451	FBgn0051451	ncRNA	-1,2	2,2E-02
NaCP60E	FBgn0085434	Na channel protein 60E	-1,3	3,2E-02
se	FBgn0086348	sepia	-1,3	4,2E-02
CG14785	FBgn0027795	CG14785 gene product from transcript CG14785-RA	-1,3	3,8E-04
CG31495	FBgn0051495	CG31495 gene product from transcript CG31495-RA	-1,3	4,7E-03
Pdp1	FBgn0016694	PAR-domain protein 1	-1,3	3,0E-02
CG1698	FBgn0033443	CG1698 gene product from transcript CG1698-RA	-1,4	4,5E-02
CG9961	FBgn0031451	CG9961 gene product from transcript CG9961-RB	-1,4	2,1E-02
l(2)34Fc	FBgn0261534	lethal (2) 34Fc	-1,4	7,8E-03
CG10804	FBgn0029663	CG10804 gene product from transcript CG10804-RD	-1,5	2,4E-02
CG4313	FBgn0025632	CG4313 gene product from transcript CG4313-RE	-1,5	1,8E-03
CG14566	FBgn0037127	CG14566 gene product from transcript CG14566-RB	-1,5	3,3E-03
Sema-1a	FBgn0011259	CG18405 gene product from transcript CG18405-RE	-1,5	9,4E-04
Faa	FBgn0016013	Fumarylacetoacetase	-1,6	1,5E-02
Obp56a	FBgn0034468	Odorant-binding protein 56a	-1,6	4,9E-02
CG1894	FBgn0039585	CG1894 gene product from transcript CG1894-RA	-1,6	5,9E-03
CG31324	FBgn0051324	CG31324 gene product from transcript CG31324-RB	-1,6	8,5E-03
CG5335	FBgn0034365	CG5335 gene product from transcript CG5335-RA	-1,6	2,8E-02
CG34002	FBgn0054002	CG34002 gene product from transcript CG34002-RB	-1,6	4,8E-04
Cyp4e2	FBgn0014469	Cytochrome P450-4e2	-1,6	1,2E-03
CG17803	FBgn0038547	CG17803 gene product from transcript CG17803-RB	-1,6	1,4E-03
pgant8	FBgn0036529	polypeptide GalNAc transferase 8	-1,6	4,9E-04
pst	FBgn0035770	pastrel	-1,6	4,1E-03
CG13692	FBgn0031254	CG13692 gene product from transcript CG13692-RA	-1,7	5,1E-03
dnc	FBgn0000479	dunce	-1,7	1,1E-02
CG31140	FBgn0051140	CG31140 gene product from transcript CG31140-RB	-1,7	1,7E-02
CG10344	FBgn0034729	CG10344 gene product from transcript CG10344-RA	-1,8	9,6E-04
CG18537	FBgn0034323	CG18537 gene product from transcript CG18537-RA	-1,9	6,7E-04
Rbm13	FBgn0030067	RNA-binding motif protein 13	-1,9	7,9E-03
tut	FBgn0052364	tumorous testis	-1,9	3,1E-04
CG3323	FBgn0029750	CG3323 gene product from transcript CG3323-RA	-1,9	2,5E-03
Cadps	FBgn0053653	Calcium-dependent secretion activator	-1,9	1,8E-04
CG3168	FBgn0029896	CG3168 gene product from transcript CG3168-RG	-2,0	1,4E-02

CG15465	FBgn0029746	CG15465 gene product from transcript CG15465-RB	-2,2	2,2E-02
Rpn13R	FBgn0029745	Regulatory particle non-ATPase 13-related	-2,4	1,3E-03
CG5455	FBgn0039430	CG5455 gene product from transcript CG5455-RA	-2,7	3,9E-05
fend	FBgn0030090	forked end	-3,1	3,9E-03
GstE1	FBgn0034335	Glutathione S transferase E1	-3,6	9,5E-05
Ac76E	FBgn0004852	Adenylyl cyclase 76E	-5,4	1,9E-04
Tektin-C	FBgn0035638	Tektin C	-6,8	4,3E-05
Syt4	FBgn0028400	Synaptotagmin 4	-7,0	4,0E-06
