# Supplementary material for: Eiger/TNFα-mediated Dilp8 and ROS production coordinate intra-organ growth in Drosophila
Source: PLoS Genet. 2019 Aug 19;15(8):e1008133. doi: 10.1371/journal.pgen.1008133 (PMC6715248; doi:10.1371/journal.pgen.1008133)
Supplement: S2 Table — (PDF) [file pgen.1008133.s008.pdf]

Gene ID	Gene Name	VDRC ID
CG11527	Tiggrin	100036
CG11853	takeout	51463
CG11853	takeout	100079
CG11062	activin-beta	108663
CG10382	wrapper	105314
CG6868	tolloid	100930
CG8807	lush	102307
CG10342	neuropeptide F	108772
CG18105	Ecdysis triggering hormone	18825
CG2040	hikaru genki	109863
CG7503	Connectin	101187
CG12410	crossveinless	106311
CG33950	terribly reduced optic lobes	24549
CG14049	dilp6	102465
CG10521	Netrin-B	100840
CG6134	spatzle	105017
CG13780	PDGF- and VEGF-related factor 2	102072
CG5619	trunk	51240
CG17579	scabrous	104703
CG8285	Boss	4366
CG1502	twisted gastrulation	108750
CG14430	boudin	107102
CG32282	dro4	103505
CG6127	Serrate	108348
CG43758	slit	108853
CG4698	Wnt oncogene analog 4	104671
CG4637	hedgehog	109454
CG12919	eiger	108814
CG32279	dro2	109207
CG8167	dilp2	102158
CG31695	screw	105303
CG8458	Wnt8	107727
CG6863	tolkin	2656
CG34378	PDGF- and VEGF-related factor 3	105008
CG1916	Wnt oncogene analog 2	104338
CG18657	Netrin-A	108577
CG10580	fringe	51977
CG13687	Prothoracicotropic hormone	102043
CG13194	pyramus	36523
CG1862	Ephrin	105139
CG30040	jelly belly	103047
CG10334	spitz	3922
CG3619	Delta	109491
CG4531	argos	47181
CG9224	short gastrulation	37407
CG15009	Ecdysone-inducible gene L2	106543
CG32268	dro6	109106
CG1171	Adipokinetic hormone-like	105063
CG14167	dilp3	106512
CG6496	Pigment-dispersing factor	50750
CG7103	PDGF- and VEGF-related factor 1	102699
CG4608	branchless	101377
CG3595	sqh	109493

CG9280	Glutactin	101918
CG15671	crossveinless 2	109915
CG2198	amalgam	22944
CG9559	folded gastrulation	101125
CG10810	Drosomycin	2703
CG4889	wingless	104579
CG6371	hugin	107771
CG5993	outstretched	3282
CG16987	dawdle	105309
CG5014	Vap-33-1	30404
CG17610	gurken	4332
CG4637	hedgehog	1403
CG13317	dilp7	105024
CG17716	faint sausage	102073
CG6588	Fasciclin I	101779
CG10812	dro5	109350
CG8561	convoluted	44361
CG5014	Vap33	100809
CG6736	dilp4	105516
CG10491	vein	109437
CG17610	gurken	101701
CG9224	short gastrulation	105853
CG13419	bursicon	102204
CG31062	sidestep	1283
CG32274	drsl	42370
CG4846	beaten path	4543
CG11326	Thrombospondin	100721
CG6407	Wnt oncogene analog 5	101621
CG2346	FMRFamide-related	103981
CG34395	nubbin	105044
CG32179	Keren	104299
CG14173	dilp1	5198
CG4700	semaphorin II	15810
CG32283	dro3	105013
CG6588	Fasciclin I	23015
CG10236	Laminin A	18873
CG12443	thisbe	102441
CG17673	Accessory gland peptide 70A	109175
CG3135	shifted	14803
