# Supplementary material for: Eiger/TNFα-mediated Dilp8 and ROS production coordinate intra-organ growth in Drosophila
Source: PLoS Genet. 2019 Aug 19;15(8):e1008133. doi: 10.1371/journal.pgen.1008133 (PMC6715248; doi:10.1371/journal.pgen.1008133)
Supplement: S3 Table — (PDF) [file pgen.1008133.s009.pdf]

DNA Damage Response pathway

ID	Symbol	Gene	logFC	P-value
FBgn0041627	Ku80	CG18801 gene product from transcript CG18801-RA	1,3	7,3E-03
FBgn0040319	Gclc	Glutamate-cysteine ligase catalytic subunit	1,0	8,5E-04
FBgn0261113	Xrp1	CG17836 gene product from transcript CG17836-RB	1,0	9,4E-04
FBgn0035996	CG3448	CG3448 gene product from transcript CG3448-RB	1,8	1,5E-03
FBgn0030506	Lig4	Ligase4	0,9	6,3E-03
FBgn0011774	Irbp	Inverted repeat-binding protein	0,9	3,9E-03

Jun-N terminal kinase pathway

ID	Symbol	Gene	logFC	P-value
FBgn0026319	Traf4	TNF-receptor-associated factor 4	1,0	2,1E-02
FBgn0036690	Ilp8	Insulin-like peptide 8	3,9	6,6E-03
FBgn0033153	Gadd45	CG11086 gene product from transcript CG11086-RA	2,3	4,0E-04
FBgn0033483	egr	eiger	1,3	1,1E-02
FBgn0030964	Pvf1	PDGF- and VEGF-related factor 1	1,1	4,6E-02

ROS/Glutatione metabolism

ID	Symbol	Gene	logFC	P-value
FBgn0063494	GstE6	Glutathione S transferase E6	3,3	1,4E-05
FBgn0010041	GstD5	Glutathione S transferase D5	2,6	4,0E-03
FBgn0033696	Cyp6g2	CG8859 gene product from transcript CG8859-RA	1,5	3,6E-03
FBgn0063493	GstE7	Glutathione S transferase E7	1,2	1,9E-04
FBgn0063491	GstE9	Glutathione S transferase E9	1,2	3,0E-03
FBgn0040319	Gclc	Glutamate-cysteine ligase catalytic subunit	1,0	8,5E-04
FBgn0086348	se	sepia	-1,3	4,2E-02
FBgn0014469	Cyp4e2	Cytochrome P450-4e2	-1,6	1,2E-03
FBgn0034335	GstE1	Glutathione S transferase E1	-3,6	9,5E-05
FBgn0040251	Ugt86Di	CG6658 gene product from transcript CG6658-RB	1,3	7,3E-04
FBgn0052196	CG32196	CG32196 gene product from transcript CG32196-RC	1,4	3,1E-03
FBgn0010038	GstD2	Glutathione S transferase D2	0,9	6,5E-02
FBgn0063495	GstE5	Glutathione S transferase E5	0,8	6,0E-02
FBgn0028396	TotA	Turandot A	3,6	1,2E-02

Ligands/secreted proteins

ID	Symbol	Gene	logFC	P-value
FBgn0036690	Ilp8	Insulin-like peptide 8	3,9	6,6E-03
FBgn0034709	Swim	Secreted Wg-interacting molecule	3,8	4,0E-05
FBgn0028396	TotA	Turandot A	3,6	1,2E-02
FBgn0033574	Spn47C	Serpin 47C	2,8	1,7E-05
FBgn0085256	CG34227	-	2,2	2,2E-03
FBgn0004629	Cys	Cystatin-like	2,1	5,2E-02
FBgn0039299	CG11854	-	2,0	5,4E-02

FBgn0031461	daw	dawdle	1,8	2,3E-01
FBgn0035092	Nplp1	Neuropeptide-like precursor 1	1,5	6,3E-02
FBgn0033483	egr	eiger	1,3	1,1E-02
FBgn0003495	spz	spätzle	1,2	6,1E-02
FBgn0034162	CG6426	-	1,1	2,7E-02
FBgn0030964	Pvf1	PDGF- and VEGF-related factor 1	1,1	4,6E-02
FBgn0043532	Obp56i	Odorant-binding protein 56i	0,9	2,8E-02
FBgn0041182	Tep2	Thioester-containing protein 2	-0,8	2,3E-02
FBgn0260660	Mp	Multiplexin	-0,9	2,5E-02
FBgn0015774	NetB	Netrin-B	-1,0	4,7E-02
FBgn0034468	Obp56a	Odorant-binding protein 56a	-1,6	4,9E-02
FBgn0054002	CG34002	-	-1,6	4,8E-04
