# Supplementary material for: Eiger/TNFα-mediated Dilp8 and ROS production coordinate intra-organ growth in Drosophila
Source: PLoS Genet. 2019 Aug 19;15(8):e1008133. doi: 10.1371/journal.pgen.1008133 (PMC6715248; doi:10.1371/journal.pgen.1008133)
Supplement: S4 Table — (PDF) [file pgen.1008133.s010.pdf]

Gen Name	Primer Fw	Primer Rv
Arc1	ACTTCTCCGCTTGCACACAC	TCCTTGTATGTCTCGATGTTGC
CG6908	GCCAACAGGTGAACGATATGG	TGGCTCGAATTTTTGCTGATCC
Hr51	GAAAGCACTACGGAATCCTAGC	CCTGGCAGCGATAAATGAGTTT
Damm	AGTGAAGGAGTGGTCTGCAAA	CAAGTGGTATTCCCTGTGGTC
Spn47C	AACAACATGCGGAATCCTGGA	TGACGTACAATCGTGTGACCA
CG15784	GGAGCGTGTTTCCCAAGAG	CCTTTTGGTGCCCTGTTCCT
mbl	TTGAATCAAAATTATAGCCCAAGCT	CGATTTTGCTCGTTAGCGTTT
CG11893	CCAGAAAACGCAGATACCGAG	ACGTCAGTAATAAACTGGGCATT
TotA	TCAACTGCTCTTATGTGCTTTGC	CTCACGATCTTCGTCGGAATAG
Cys	TGTATTTTGGGTCTGGTTCTCG	CTCCTTCCTGCTGTTTCCCTC
CG11854	AAAGCGGTATCAAGGAGCTGG	GATTGCGTCCGATTTTGAACTTC
mmp1	TCAGTGTTCATAGTCGTAGGCA	GGTAGATAGCCGAACTGGGAC
rpr	TGGCATTCTACATACCCGATCA	CCAGGAATCTCCACTGTGACT
egr	CAGCTGATCCCCCTGGTTTT	GCCAGATCGTTAGTGCGAGA
Ku80	ATGGCTTCTAACAAGGAATGCC	AGAATCTCCGCCACACATTTTG
Xrp1	GTATCGACATCAACCAGGAGC	GCGAGATAGGGGTCCTCTGA
Corp	CAGCTTGAAGGTTCTCTTCCAG	GGGAGATGACCTCTTCGTAAGT
dilp8	ACGCACAACAAGCATCACTAC	AGTTCGCTGAGGCGATTGAAG
Nlaz	ACGCCAACTACAGTCTCATAGA	CGAGGGTTGTCCGGTGAATC
upd3	ATCCCCTGAAGCACCTACAGA	CAGTCCAGATGCGTACTGCTG
daw	CGGTTCCAGGTGTTTCAGC	ATCCTTCGTCCGCATCCTAAG
puc	AGC GAT ACG CCA CAT CAG AAC	ACT TGT ACC GCA TGA CGT AGG
upd2	TCTTCTGCTGATCCTTGCGGAACT	AGCTAAAGACTTGGTACCGCCACA
egr.prom	TTTCCCTCAGTTCACGCTCC	CTTCGCTATCATGCACGCAC
rpr.prom	TGTCAGGTTGGTTCTTCCACT	GTCGATGCACGCTGAGTGA
egr.3UTR	TCGCCAGCGTGAATCCATTA	CACGCTTACACAAGCGGGTA
egr.I1	CTCCAAAAACCATCACAACGACA	CTAGAAGGCGGTTCATGGCT
